# Pleiotropic and Sex-Specific Effects of Cancer GWAS SNPs on Melanoma Risk in the Population Architecture Using Genomics and Epidemiology (PAGE) Study

**DOI:** 10.1371/journal.pone.0120491

**Published:** 2015-03-19

**Authors:** Jonathan M. Kocarnik, S. Lani Park, Jiali Han, Logan Dumitrescu, Iona Cheng, Lynne R. Wilkens, Fredrick R. Schumacher, Laurence Kolonel, Chris S. Carlson, Dana C. Crawford, Robert J. Goodloe, Holli H. Dilks, Paxton Baker, Danielle Richardson, Tara C. Matise, José Luis Ambite, Fengju Song, Abrar A. Qureshi, Mingfeng Zhang, David Duggan, Carolyn Hutter, Lucia Hindorff, William S. Bush, Charles Kooperberg, Loic Le Marchand, Ulrike Peters

**Affiliations:** 1 Division of Public Health Sciences, Fred Hutchinson Cancer Research Center, Seattle, Washington, United States of America; 2 Department of Preventive Medicine, Norris Comprehensive Cancer Center, Keck School of Medicine, University of Southern California, Los Angeles, California, United States of America; 3 Department of Epidemiology, Richard M. Fairbanks School of Public Health, Melvin and Bren Simon Cancer Center, Indiana University, Indianapolis, Indiana, United States of America; 4 Center for Human Genetics Research, Vanderbilt University, Nashville, Tennessee, United States of America; 5 Department of Molecular Physiology and Biophysics, Vanderbilt University, Nashville, Tennessee, United States of America; 6 Cancer Prevention Institute of California, Fremont, California, United States of America; 7 Epidemiology Program, University of Hawaii Cancer Center, Honolulu, Hawaii, United States of America; 8 Department of Epidemiology, Case Western Reserve University, Cleveland, Ohio, United States of America; 9 Biostatistics Institute for Computational Biology, Case Western Reserve University, Cleveland, Ohio, United States of America; 10 Department of Genetics, Rutgers University, Piscataway, New Jersey, United States of America; 11 Information Sciences Institute, University of Southern California, Marina del Rey, California, United States of America; 12 Department of Epidemiology, Tianjin Medical University Cancer Institute and Hospital, Tianjin, People’s Republic of China; 13 Channing Laboratory, Department of Medicine, Brigham and Women’s Hospital, Harvard Medical School, Boston, Massachusetts, United States of America; 14 Department of Dermatology, Brigham and Women’s Hospital, Boston, Massachusetts, United States of America; 15 Translational Genomics Research Institute, Phoenix, Arizona, United States of America; 16 Epidemiology and Genomics Research Program, Division of Cancer Control and Population Sciences, NCI, NIH, Bethesda, Maryland, United States of America; 17 Division of Genomic Medicine, NHGRI, NIH, Bethesda, Maryland, Untied States of America; 18 Department of Biomedical Informatics, Vanderbilt University, Nashville, Tennessee, United States of America; Nanjing Medical University, CHINA

## Abstract

**Background:**

Several regions of the genome show pleiotropic associations with multiple cancers. We sought to evaluate whether 181 single-nucleotide polymorphisms previously associated with various cancers in genome-wide association studies were also associated with melanoma risk.

**Methods:**

We evaluated 2,131 melanoma cases and 20,353 controls from three studies in the Population Architecture using Genomics and Epidemiology (PAGE) study (EAGLE-BioVU, MEC, WHI) and two collaborating studies (HPFS, NHS). Overall and sex-stratified analyses were performed across studies.

**Results:**

We observed statistically significant associations with melanoma for two lung cancer SNPs in the *TERT-CLPTM1L* locus (Bonferroni-corrected p<2.8x10^-4^), replicating known pleiotropic effects at this locus. In sex-stratified analyses, we also observed a potential male-specific association between prostate cancer risk variant rs12418451 and melanoma risk (OR=1.22, p=8.0x10^-4^). No other variants in our study were associated with melanoma after multiple comparisons adjustment (p>2.8e^-4^).

**Conclusions:**

We provide confirmatory evidence of pleiotropic associations with melanoma for two SNPs previously associated with lung cancer, and provide suggestive evidence for a male-specific association with melanoma for prostate cancer variant rs12418451. This SNP is located near *TPCN2*, an ion transport gene containing SNPs which have been previously associated with hair pigmentation but not melanoma risk. Previous evidence provides biological plausibility for this association, and suggests a complex interplay between ion transport, pigmentation, and melanoma risk that may vary by sex. If confirmed, these pleiotropic relationships may help elucidate shared molecular pathways between cancers and related phenotypes.

## Introduction

As the most serious form of skin cancer, melanoma is a considerable public health burden. In 2013, there were an estimated 76,690 new diagnoses and 9,480 deaths from melanoma in the United States alone [1]. Ultraviolet (UV) radiation exposure is the largest environmental risk factor for melanoma, with an estimated 44–90% of melanoma attributable to sun exposure [2]. Other risk factors include artificial UV sources such as tanning beds [3], larger numbers of nevi, pigmentation traits (light versus dark hair, eye, and skin color), race/ethnicity (European versus non-European ancestry), skin response to UV exposure (burn versus tan), older age, and male sex [2]. Anatomic location of melanoma also tends to vary by sex, arising most commonly on the back, abdomen, and chest in males, and on the lower leg, hip, and thigh in females [2]. Females also appear to have lower risk of metastases and longer melanoma-specific survival than males [4]. In addition to environmental exposures, genetic risk factors have also been implicated for both familial and sporadic disease. Genome-wide association studies (GWAS) have successfully identified at least 11 susceptibility loci for melanoma [5, 6].

Several cancer susceptibility loci identified in GWAS, such as the 8q24 and *TERT-CLPTM1L* loci, have also been associated with numerous other cancer sites [7, 8]. Variants in the *TERT-CLPTM1L* region, for example, have been associated with basal cell carcinoma, melanoma, and glioma, as well as lung, bladder, prostate, pancreatic, and cervical cancers [8–10]. This provides evidence of pleiotropy, where a single genotype or locus is associated with multiple phenotypes. The existence of such pleiotropic effects suggests that there may be common mechanisms of carcinogenesis or disease susceptibility pathways across cancer phenotypes. Finding these effects can be useful for elucidating pathogenic mechanisms, improving disease classification, or targeting therapeutic intervention. While identifying and characterizing pleiotropy is important, the extent of pleiotropy has not been comprehensively explored. This study aims to evaluate single nucleotide polymorphisms (SNPs) associated with various cancers in previous GWAS for additional pleiotropic associations with melanoma. As melanoma risk and anatomic location have been shown to vary by sex [2], this study also evaluates whether any of these genetic associations may vary by sex as well.

## Material and Methods

### Study Populations

We analyzed 2,131 melanoma cases and 20,353 melanoma-free controls from five study populations. Three of these studies collaborated through their participation in the Population Architecture using Genomics and Epidemiology (PAGE) Study [11]: the Multiethnic Cohort (MEC) [12]; the Women’s Health Initiative (WHI) [13]; and Epidemiological Architecture for Genes Linked to Environment (EAGLE), accessing BioVU, the Vanderbilt biorepository linked to de-identified electronic medical records [14, 15]. Two non-PAGE studies also contributed: the Nurses’ Health Study (NHS) [16, 17] and the Health Professionals Follow-up Study (HPFS) [18]. NHS and WHI are female-only studies, and HPFS is a male-only study. Additional details on each of these studies are provided in the Supplemental Material (S1 File).

Each study performed a nested case-control analysis of melanoma using a subset of their overall study population. Invasive melanoma cases were defined as incident cases of melanoma in participants without a previous cancer diagnosis (except for non-melanoma skin cancer). EAGLE-BioVU also included prevalent melanoma cases diagnosed up to 5 years before entrance into the study, and some cases could have had prior cancers. Incident cancers were identified through follow-up questionnaires (WHI, NHS, HPFS), tumor and cancer surveillance registries (EAGLE-BioVU, MEC) and medical record entries (EAGLE-BioVU). Melanoma in situ cases were excluded in all studies.

Both matched and unmatched melanoma-free controls were used. In PAGE, a subset of controls were matched to melanoma cases on age (EAGLE-BioVU, MEC, WHI), sex (EAGLE-BioVU, MEC), enrollment date (WHI), race/ethnicity (EAGLE-BioVU, MEC, WHI), randomization arm (WHI), study site (MEC), or blood/urine collection factors (MEC). To improve power, each PAGE study also utilized additional unmatched melanoma-free controls, which had been matched to cases of other cancer types for similar PAGE analyses (sensitivity analyses showed no difference from including these additional controls). NHS and HPFS controls were not matched to melanoma cases, but came from previously matched nested case-control GWAS. Demographic and epidemiologic data were obtained according to individual study protocols. Due to low case numbers in other race/ethnicity groups, we restricted our analysis to participants of European ancestry.

The protocol for this study was approved by Institutional Review Boards at their respective institutions: BioVU was approved by the Vanderbilt Institutional Review Board; HPFS and NHS were approved by the Institutional Review Board at Brigham and Women’s Hospital and the Harvard School of Public Health; MEC was approved by the Human Studies Program at the University of Hawaii and the Office for the Protection of Research Subjects at the University of Southern California; and WHI was approved by the Fred Hutchinson Cancer Research Center Institutional Review Board. All participants of HPFS, NHS, MEC, and WHI provided written informed consent. All BioVU participants signed a ‘‘consent-to-treatment” form informing them that anonymized genetic information from their discarded blood, along with de-identified EMR information, would be used for research; participants were given the choice to decline participation via an ‘‘opt-out” box on the form.

### SNP Selection and Genotyping

In PAGE, a custom panel of 189 SNPs associated with risk of various cancer types was selected and genotyped. SNPs were chosen based on the literature as of 2010 [11], as well as SNPs associated with cancer in the National Human Genome Research Institute GWAS catalog [19]. Each PAGE study genotyped a subset of this panel in order to maximize replication and generalization opportunities according to the characteristics of their study population. The risk allele for each SNP was defined as the allele associated with an increased risk of cancer, based on prior literature for the first reported association (S1 Table). Eight of these SNPs were originally identified as associated with melanoma risk, and were analyzed separately [20]. Several SNPs included in the PAGE panel were later reported to be associated with additional cancers, including melanoma. To remain consistent with their original reason for inclusion in the pleiotropy analyses, we analyzed all SNPs according to their initially reported cancer association. Thus, 181 SNPs were evaluated in this analysis.

Standard quality assurance and quality control measures were utilized to ensure genotyping quality. In PAGE, samples and SNPs were included based on call rates (≥90%), concordance of blinded replicates (>98%), and no strong evidence of departure from Hardy-Weinberg equilibrium expectations (p<0.001). Each laboratory also genotyped 360 HapMap samples to serve as cross-laboratory and cross-platform quality control samples [21].

In NHS and HPFS, participants had been previously genotyped in nested case-control GWAS of various outcomes (S1 File). For the melanoma GWAS, >2.5 million SNPs were imputed based on NCBI build 35 of phase II HapMap CEU data using MACH. Only SNPs with an imputation quality r^2^>0.95 in each study were included. Genotype information for the panel of 189 SNPs assembled by PAGE was available from this existing GWAS data.

To evaluate the pairwise correlation between SNPs in a region (such as *TPCN2*), we used the program SNAP [22]. As our study was restricted to those of European ancestry, we used the 1000 Genomes Pilot CEU data for obtaining r^2^ values between SNPs.

### Statistical Analyses

For each study we estimated the association between individual SNPs and risk of melanoma using unconditional logistic regression. SNPs were coded additively with 0, 1, 2 referring to the number of purported risk alleles (or the dosage for imputed SNPs), defined as the allele that increased the risk of cancer in the initial GWAS publication. Models were adjusted for age (all studies) and sex (EAGLE-BioVU and MEC only). In NHS and HPFS, models were also adjusted for each study’s five most-significant GWAS-derived eigenvectors, using EIGENSTRAT [23], to account for population substructure. The three PAGE studies used ancestry informative markers to identify continental genetic ancestry of participants [24]. Since participants were already restricted to those of European ancestry, and GWAS-derived markers were not available, we did not adjust for principal components in these three studies.

Study-specific regression estimates were combined across studies using inverse-variance weighted fixed-effect meta-analysis. We calculated the heterogeneity p-values based on Cochran’s Q statistic. Analyses were performed using Stata version 12 [25]. Because of multiple testing, we used a Bonferroni-corrected p-value threshold to determine the statistical significance of the overall association for each SNP with melanoma (p<0.05/181 = 2.8x10^-4^). In order to evaluate for potential sex-specific genetic effects, we also evaluated the association between each SNP and melanoma risk stratified by sex. We performed meta-regression to obtain p-heterogeneity values for the difference between sex-specific regression estimates, using a significance threshold of p-heterogeneity<0.05.

## Results

Demographic and epidemiologic characteristics of the study populations are provided in Table 1. Since NHS and WHI are female-only studies, the overall analysis included roughly twice as many females as males. Melanoma cases were generally of similar age as controls (overall mean age of 65 in cases vs. 63 in controls).

**Table 1:** Demographic characteristics of the five studies contributing to this analysis.

	Study	EAGLE-BioVU		HPFS		MEC		NHS		WHI		Total	
Characteristic		Cases	Controls	Cases	Controls	Cases	Controls	Cases	Controls	Cases	Controls	Cases	Controls
# Participants		742	8,063	177	2,251	240	2,032	317	3,377	655	4,630	2,131	20,353
Sex	Males	445	4,351	177	2,251	149	1,059	0	0	0	0	771	7,661
	Females	297	3,712	0	0	91	973	317	3,377	655	4,630	1,360	12,692
Age	Mean	64	56	61	61	67	69	57	57	69	77	65	63
	SD	12.4	15.6	9.3	8.5	9.5	8.5	6.8	6.7	7.1	7.1	11.0	14.4

**Fig 1:**
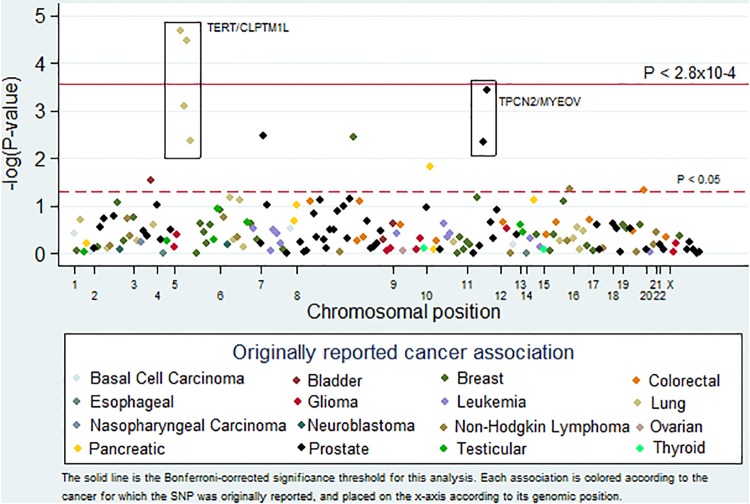
Pleiotropy-colored Manhattan plot. This plot shows the inverse log of the P-value for the association between melanoma and SNPs previously associated with cancer. The solid line represents the Bonferroni-corrected significance threshold for this analysis (0.05/181 = 2.8x10^-4^). Each association is colored according to the cancer for which the SNP was originally reported, and placed on the x-axis according to its genomic position.

In total we evaluated 181 cancer GWAS SNPs for an association with melanoma (Fig. 1). Two SNPs were statistically significantly associated with melanoma: rs4975616 and rs401681, both in the *TERT/CLPTM1L* locus (Odds Ratio (OR) = 0.87, 95% Confidence Interval (CI): 0.81–0.93, p-values < 3.7x10^-5^, Table 2a). Both of these SNPs were originally identified in GWAS of lung cancer, and then later additionally associated with melanoma [8, 26]. Of note, our results are consistent with previous studies showing that these SNPs have pleiotropic effects in opposite directions for different cancer types. Specifically, for both SNPs the allele associated with an increased risk of lung cancer appears to also be associated with a decreased risk of melanoma.

**Table 2:** Association between cancer GWAS SNPs and melanoma.

**Table 2a)**									
SNP	Gene	Chromosome / Risk allele	Original cancer association	n	# Studies	OR	95% CI	p-value	p-hetero-geneity
rs4975616	*TERT/CLPTM1L*	5 / A	Lung cancer	22,135	5	0.87	(0.81–0.93)	**2.30E-05**	0.78
rs401681	*TERT/CLPTM1L*	5 / C	Lung cancer	22,109	5	0.87	(0.81–0.93)	**3.65E-05**	0.70

**Table 2b)**									
SNP	Gene	Chromosome / Risk allele	Original cancer association	n	# Studies	OR	95% CI	p-value	p-hetero-geneity
rs7117034	*TPCN2*, *MYEOV* (near)	11 / T	Prostate cancer	10,675	2	1.23	(1.10–1.37)	3.67E-04	0.55
rs402710	*CLPTM1L* (intronic)	5 / C	Lung cancer	15,991	3	0.87	(0.81–0.94)	7.74E-04	0.04
rs12155172	*ABCB5* (near)	7 / A	Prostate cancer	10,703	2	1.19	(1.06–1.33)	3.38E-03	0.61
rs13281615	*MYC*, *POU5F1B* (near)	8 / T	Breast cancer	22,138	5	1.11	(1.03–1.18)	3.44E-03	0.20
rs31489	*CLPTM1L* (intronic)	5 / C	Lung cancer	14,004	2	0.89	(0.82–0.96)	4.18E-03	0.33
rs12418451	*TPCN2*, *MYEOV* (near)	11 / A	Prostate cancer	22,053	5	1.11	(1.03–1.19)	5.03E-03	0.30
rs505922	*ABO* (intronic)	9 / C	Pancreatic cancer	13,339	4	0.89	(0.82–0.98)	0.013	0.40
rs710521	*LEPREL1*, *TP63* (near)	3 / A	Urinary bladder cancer	9,608	3	1.13	(1.01–1.27)	0.028	0.55
rs7176508	*RPLP1*, *GEMIN8P1* (near)	15 / A	Chronic lymphocytic leukemia	11,395	3	1.10	(1.00–1.21)	0.039	0.78
rs10411210	*RHPN2* (intronic)	19 / C	Colorectal cancer	15,971	3	1.14	(1.00–1.30)	0.046	0.68

2a) SNPs showing a statistically significant association with melanoma at a Bonferroni-corrected threshold of 0.05/181 = 2.8x10^-4^.

2b) SNPs showing a marginal association with melanoma at p<0.05.

No other SNP was associated with melanoma below our Bonferroni-corrected statistical significance threshold of p<2.8x10^-4^, though 10 additional SNPs had p-values below 0.05 (Table 2b). These SNPs were previously associated with seven different cancers. Seven of these ten SNPs showed an increased risk for melanoma in the same direction as the previously associated cancer (OR = 1.10–1.23). The other three of these SNPs showed a decreased risk of melanoma: two *TERT/CLPTM1L* SNPs previously associated with lung cancer (rs402710, OR = 0.87; rs31489, OR = 0.89) and one *ABO* SNP previously associated with pancreatic cancer (rs505922, OR = 0.89). Due to multiple testing, some (or all) of these marginal findings could be due to chance (expect 0.05*181 = 9.05), though correlated SNPs may not represent independent tests. Results for all 181 SNPs are provided in S2 Table.

In the sex-stratified analyses, one additional SNP, rs12418451 (near *TPCN2*), nearly reached statistical significance in males (p = 7.96x10^-4^), but not in females or overall (p-heterogeneity = 0.04, Table 3a), and also showed a larger effect in males (OR = 1.22, 95% CI: 1.09–1.37) than females (OR = 1.05, 95% CI: 0.96–1.14). Four other nearby (40–60kb away) but uncorrelated (r^2^ with rs12418451 < 0.2) SNPs in this region also showed a trend of stronger effects in males than females, though none suggested a statistically significant difference (p-heterogeneity>0.05, Table 3b). In total, 12 additional SNPs (13 total including rs12418451) showed between-sex heterogeneity p-values below 0.05, slightly more than we expected by chance (0.05*181 = 9.05), but the association was not significant in either sex stratum. Sex-stratified results for all SNPs are provided in S3 Table.

**Table 3:** Association between SNPs in the *TPCN2/MYEOV* region and melanoma.

Table 3a)								p-heterogeneity	
SNP	Gene, Chromosome / coded allele, Previous trait	Sex	# Studies	n	OR	95% CI	p-value	Between-studies	Between-sexes
rs12418451	*TPCN2*, *MYEOV* (near)	Combined	5	22,053	1.11	(1.03–1.19)	5.03E-03	0.30	
	11 / A	Male	3	8,213	1.22	(1.09–1.37)	7.96E-04	0.21	**0.04**
	Prostate cancer	Female	4	13,840	1.05	(0.96–1.14)	0.33	0.42	

**Table 3b)**								p-heterogeneity	
SNP /r^2^ with rs12418451	Gene, Chromosome / coded allele, Previous trait	Sex	# Studies	n	OR	95% CI	p-value	Between-studies	Between-sexes
rs11228565	*TPCN2*, *MYEOV* (near)	Combined	2	14,026	1.06	(0.96–1.17)	0.22	4.38E-04	
r^2^ = 0.13	11 / A	Male	1	4,756	1.21	(1.03–1.42)	0.02	.	0.051
	Prostate cancer	Female	2	9,270	0.99	(0.87–1.12)	0.84	3.53E-03	
rs10896449	*TPCN2*, *MYEOV* (near)	Combined	5	22,092	1.05	(0.99–1.12)	0.12	0.53	
r^2^ = 0.07	11 / G	Male	3	8,221	1.10	(0.99–1.23)	0.07	0.86	0.28
	Prostate cancer	Female	4	13,871	1.02	(0.94–1.11)	0.58	0.34	
rs7117034	*TPCN2*, *MYEOV* (near)	Combined	2	10,675	1.23	(1.1–1.37)	3.67E-04	0.55	
r^2^ = 0.13	11 / T	Male	2	5,775	1.26	(1.09–1.45)	2.07E-03	0.20	0.62
	Prostate cancer	Female	2	4,900	1.18	(0.99–1.42)	0.07	0.44	
rs7931342	*TPCN2*, *MYEOV* (near)	Combined	4	13,336	1.03	(0.95–1.12)	0.48	0.68	
r^2^ = 0.05	11 / G	Male	2	3,451	1.05	(0.89–1.25)	0.56	0.76	0.78
	Prostate cancer	Female	3	9,885	1.02	(0.93–1.13)	0.63	0.37	

3a) Association between SNP rs12418451 melanoma, both overall and stratified by sex.

3b) Four additional SNPs within the same locus as rs12418451, shown for comparison (r^2^ with rs12418451 < 0.13 in 1000G CEU).

## Discussion

We replicated previously reported associations with melanoma for two SNPs in the *TERT-CLPTM1L* region. These SNPs were previously shown to demonstrate pleiotropic effects in opposing directions, with decreased risk for melanoma but increased risk for lung and other cancers. We also observed a marginally significant association with melanoma in *TPCN2*, suggesting a potential male-specific pleiotropic association with both melanoma and prostate cancer. Notably, none of the other cancer susceptibility SNPs evaluated showed evidence for a pleiotropic association with melanoma.

The two SNPs demonstrating a significant association with melanoma (rs401681 and rs4975616) are located in the *TERT-CLPTM1L* locus, which contains variants associated with a number of different cancers. The pleiotropic effects of variants in this region have been well established [8–10], and our findings are consistent with previous reports associating cancer risk variants in this region with decreased risk of melanoma [8, 26, 27]. Specifically, the *C* allele of rs401681 has been associated with an increased risk of lung cancer [28], basal cell carcinoma [27], bladder cancer, prostate cancer, and cervical cancer [8]. This same allele has also been associated with a decreased risk of melanoma [8, 27] and pancreatic cancer [29]. The other SNP in this region that was statistically significant in our study, rs4975616 (*A* allele), has also been previously associated with an increased risk of lung cancer [28, 30] and a decreased risk of melanoma [26]. While not reaching our Bonferroni cutoff, two other SNPs in this region were also marginally associated with decreased risk of melanoma in our study: rs402710 (*C* allele, p = 7.74x10^-4^) and rs31489 (*C* allele, p = 4.18x10^-3^). These alleles have also been previously associated with increased risk of lung cancer [9], as well as increased risk of bladder cancer (rs402710) and decreased risk of testicular or pancreatic cancer (rs31489) [10]. These four SNPs are all located within the *CLPTM1L* gene and are in relatively high linkage disequilibrium with each other (r^2^>0.57; from 1000 Genomes Project pilot CEU data using SNAP [22]). Two nearby SNPs within the *TERT* gene were not associated with melanoma (p>0.39), and were not correlated with any of the four *CLPTM1L* SNPs (r^2^<0.07). Taken together, our findings provide further evidence of pleiotropic effects in opposite directions in the *TERT-CLPTM1L* region, where variants associated with increased risk for lung and other cancers are simultaneously associated with reduced melanoma risk.

In our sex-stratified analyses, we identified one SNP (rs12418451) that demonstrated a marginally significant association with melanoma in males, but not in either the female or overall analyses. Previously associated with prostate cancer [31], this SNP is located ~77kb downstream of *TPCN2* and ~126kb upstream of *MYEOV*. The proximity of this SNP to these other genes provides biological plausibility for an association with melanoma. The nearby *TPCN2* (two-pore segment channel 2) encodes a putative cation-selective ion channel that releases Ca^2+^ from acidic organelles [32]. Similarly to other ion transport genes associated with melanoma, such as *SLC45A2*, variants in *TPCN2* may impact melanogenesis through pH regulation [33]. Indeed, two coding variants in *TPCN2* have been associated with pigmentation traits (blond versus brown hair color [34]), though neither are highly correlated with rs12418451 (r^2^<0.07). A later study did not find either of these two *TPCN2* SNPs to be associated with melanoma (p>0.12), though they did not stratify by sex [35].

SNP rs12418451 is also ~126 kb upstream of *MYEOV*, an oncogene that includes variants implicated for multiple cancers, including multiple myeloma, breast cancer, colon cancer, and esophageal squamous cell carcinoma [36]. A proxy of rs12418451 is also one of three independent loci in this region associated with prostate cancer [37]. Another study evaluating this region for prostate cancer identified an interaction between rs12418451 and rs784411 in *CEP152*, a centrosomal protein shown to function as a regulator of genomic integrity and cellular response to DNA damage [36]. In our study, a second SNP in this region (rs7117034, ~117kb downstream of *TPCN2*) was also marginally associated with melanoma risk overall (p = 3.7x10^-4^). While this SNP also suggested a stronger effect in males than females (OR = 1.26 and 1.18, respectively), this difference was not statistically significant (p-heterogeneity = 0.62). Together, our findings identify a potentially novel pleiotropic finding for a sex-specific association between rs12418452 and melanoma, and highlight a new locus for melanoma with plausible biologic function.

Our sex-specific finding for SNP rs12418451 also raises interesting questions regarding potential sex differences in the relationships between ion transport, pigmentation, and melanoma. We recently reported a sex difference in association with melanoma for rs16891982 in *SLC45A2*, another SNP in a solute-carrier gene associated with pigmentation [20]. These and other melanosome ion transporter proteins have demonstrated the functional importance of ion and small molecule transport to melanogenesis and the pigmentation pathway [38, 39]. Though they transport different molecules, these SNPs in *SLC45A2* and near *TPCN2* both demonstrated associations with melanoma that were larger in males than in females.

Previous evidence that skin pigmentation processes can be up- or down-regulated by sex hormones provides biological plausibility for such a difference. Findings in a study of the hyperpigmentation condition melasma, for example, supported the role of several ion transporters in the estrogen-induced expression of tyrosinase [40]. Another study found that androgens can inhibit tyrosinase activity [41]. As the rate-limiting enzyme in melanin synthesis, the regulation of tyrosinase activity impacts skin pigmentation through the levels of eumelanin and pheomelanin produced [33]. As males and females differ in their circulating levels of sex hormones, it is feasible that hormones impact ion exchange or tyrosinase activity in a way that modifies the effect of these variants on melanoma risk, perhaps through alterations to melanogenesis or skin pigmentation. As such, variants in other ion transport genes similar to *TPCN2* and *SLC45A2* might also be expected to impact pigmentation and melanoma risk. Interestingly, sex differences in the genetic effect of solute carrier genes have also been seen for other phenotypes, such as *LYPLAL1/SLC30A10* with waist-hip ratio [42]. While suggestive, further research to evaluate these potential sex differences for melanoma risk is needed.

The strengths of this study stem from the collaboration of five large studies, which together provide sizable samples to evaluate the association of melanoma with cancer GWAS SNPs. A potential limitation is that three of these studies were conducted only in males (HPFS) or females (NHS, WHI). Since not all SNPs were available in all studies, sample sizes also varied by SNP depending on which studies had that particular SNP available. These differences in sample size may have reduced our ability to detect an association with melanoma for some SNPs. However, 97% of SNPs were available in at least two studies (and 75% in at least three), and most overall analyses were large (mean number of participants available per SNP 14,836, range 1,925–22,141). An additional limitation is that we were unable to test whether some of our findings are independently associated with melanoma, or are due to an association with pigmentation characteristics. Unfortunately, data were not available to evaluate these associations according to skin/hair pigmentation or anatomical location. Additional work will be needed to explore the relationships between these genetic variants, pigmentation characteristics, and melanoma.

## Conclusions

In summary, we provided confirmatory evidence of pleiotropic associations with melanoma for two SNPs in *TERT-CLPTM1L* and identified a potentially novel sex-specific association for a SNP near *TPCN2/MYEOV*. Variants in the *TERT-CLPTM1L* locus demonstrated pleiotropic effects in opposite directions from other cancers, where the allele previously associated with increased risk of lung and other cancers demonstrated an association with decreased risk of melanoma in our study. Additionally, we were able to provide some evidence of an association with melanoma for one SNP near solute-carrier gene *TPCN2* that showed potential differences in effect by sex, with a larger effect in males than females. Previously associated with increased risk of prostate cancer, this SNP demonstrated a potentially pleiotropic effect of increased risk of melanoma. While this latter finding did not reach statistical significance, it is a biologically plausible candidate for follow-up studies.

## Supporting Information

S1 File Study descriptions. Detailed descriptions for each of the five studies contributing to this analysis.
(DOCX)

S1 Table Full list of 181 SNPs evaluated for an association with melanoma. Provides the allele associated with increased risk in the original cancer GWAS publication, as well as the chromosomal location of the SNP and gene.
(DOCX)

S2 Table Full results. Results for the association between melanoma and each of the 181 SNPs.
(DOCX)

S3 Table Full sex-stratified results. Results for the association between melanoma and each of the 181 SNPs, stratified by sex.
(DOCX)

## References

[pone.0120491.ref001] 1. Howlader N, Noone AM, Krapcho M, Garshell J, Neyman N, Altekruse SF, et al. SEER Cancer Statistics Review, 1975–2010. National Cancer Institute, Bethesda, MD2013 [based on November 2012 SEER data submission, posted to the SEER website, April 2013]; Available from: http://seer.cancer.gov/csr/1975_2010/.

[2] Gruber SB, Armstrong BK. Cutaneous and ocular melanoma In: Schottenfeld D, Fraumeni JF, editors. Cancer Epidemiology and Prevention. 3 ed New York, NY, USA: Oxford University Press; 2006 p. 1126–229.

[3] Boniol M, Autier P, Boyle P, Gandini S. Cutaneous melanoma attributable to sunbed use: systematic review and meta-analysis. BMJ. 2012;345: e4757 10.1136/bmj.e4757 22833605 PMC3404185

[4] Joosse A, de Vries E, Eckel R, Nijsten T, Eggermont AM, Holzel D, et al Gender differences in melanoma survival: female patients have a decreased risk of metastasis. J Invest Dermatol. 2011;131(3): 719–26. 10.1038/jid.2010.354 21150923

[5] Gerstenblith MR, Shi J, Landi MT. Genome-wide association studies of pigmentation and skin cancer: a review and meta-analysis. Pigment Cell Melanoma Res. 2010;23(5): 587–606. 10.1111/j.1755-148X.2010.00730.x 20546537 PMC3179913

[6] Barrett JH, Iles MM, Harland M, Taylor JC, Aitken JF, Andresen PA, et al Genome-wide association study identifies three new melanoma susceptibility loci. Nat Genet. 2011;43(11): 1108–13. 10.1038/ng.959 21983787 PMC3251256

[7] Ghoussaini M, Song H, Koessler T, Al Olama AA, Kote-Jarai Z, Driver KE, et al Multiple loci with different cancer specificities within the 8q24 gene desert. J Natl Cancer Inst. 2008;100(13): 962–6. 10.1093/jnci/djn190 18577746 PMC2902819

[8] Rafnar T, Sulem P, Stacey SN, Geller F, Gudmundsson J, Sigurdsson A, et al Sequence variants at the TERT-CLPTM1L locus associate with many cancer types. Nat Genet. 2009;41(2): 221–7. 10.1038/ng.296 19151717 PMC4525478

[9] Baird DM. Variation at the TERT locus and predisposition for cancer. Expert Rev Mol Med. 2010;12: e16 10.1017/S146239941000147X 20478107

[10] Mocellin S, Verdi D, Pooley KA, Landi MT, Egan KM, Baird DM, et al Telomerase reverse transcriptase locus polymorphisms and cancer risk: a field synopsis and meta-analysis. J Natl Cancer Inst. 2012;104(11): 840–54. 10.1093/jnci/djs222 22523397 PMC3611810

[11] Matise TC, Ambite JL, Buyske S, Carlson CS, Cole SA, Crawford DC, et al The Next PAGE in understanding complex traits: design for the analysis of Population Architecture Using Genetics and Epidemiology (PAGE) Study. Am J Epidemiol. 2011;174(7): 849–59. 10.1093/aje/kwr160 21836165 PMC3176830

[12] Kolonel LN, Henderson BE, Hankin JH, Nomura AM, Wilkens LR, Pike MC, et al A multiethnic cohort in Hawaii and Los Angeles: baseline characteristics. Am J Epidemiol. 2000;151(4): 346–57. 10695593 10.1093/oxfordjournals.aje.a010213 PMC4482109

[13] Hays J, Hunt JR, Hubbell FA, Anderson GL, Limacher M, Allen C, et al The Women's Health Initiative recruitment methods and results. Ann Epidemiol. 2003;13(9 Suppl): S18–77. 14575939 10.1016/s1047-2797(03)00042-5

[14] Bush W, Boston J, Pendergrass S, Dumitrescu L, Goodloe R, Brown-Gentry K, et al Enabling high-throughput genotype-phenotype associations in the Epidemioloic Architecture for Genes Linked to Environment (EAGLE) project as part of the Populations Architecture using Genomics and Epidemiology (PAGE) study. Pac Symp Biocomput. 2013(18): 373–84. 23424142 PMC3579641

[15] Roden DM, Pulley JM, Basford MA, Bernard GR, Clayton EW, Balser JR, et al Development of a large-scale de-identified DNA biobank to enable personalized medicine. Clin Pharmacol Ther. 2008;84(3): 362–9. 10.1038/clpt.2008.89 18500243 PMC3763939

[16] Belanger CF, Hennekens CH, Rosner B, Speizer FE. The nurses' health study. Am J Nurs. 1978;78(6): 1039–40. 248266

[17] Colditz GA, Hankinson SE. The Nurses' Health Study: lifestyle and health among women. Nat Rev Cancer. 2005;5(5): 388–96. 15864280 10.1038/nrc1608

[18] Rimm EB, Stampfer MJ, Colditz GA, Chute CG, Litin LB, Willett WC. Validity of self-reported waist and hip circumferences in men and women. Epidemiology. 1990;1(6): 466–73. 2090285 10.1097/00001648-199011000-00009

[19] Hindorff LA, Sethupathy P, Junkins HA, Ramos EM, Mehta JP, Collins FS, et al Potential etiologic and functional implications of genome-wide association loci for human diseases and traits. Proc Natl Acad Sci U S A. 2009;106(23): 9362–7. 10.1073/pnas.0903103106 19474294 PMC2687147

[20] Kocarnik JM, Park SL, Han J, Dumitrescu L, Cheng I, Wilkens LR, et al Replication of associations between GWAS SNPs and melanoma risk in the Population Architecture Using Genomics and Epidemiology (PAGE) Study. J Invest Dermatol. 2014;134(7): 2049–52. 10.1038/jid.2014.53 24480881 PMC4057959

[21] International HapMap Consortium. The International HapMap Project. Nature. 2003;426(6968): 789–96. 14685227 10.1038/nature02168

[22] Johnson AD, Handsaker RE, Pulit SL, Nizzari MM, O'Donnell CJ, de Bakker PI. SNAP: a web-based tool for identification and annotation of proxy SNPs using HapMap. Bioinformatics. 2008;24(24): 2938–9. 10.1093/bioinformatics/btn564 18974171 PMC2720775

[23] Price AL, Patterson NJ, Plenge RM, Weinblatt ME, Shadick NA, Reich D. Principal components analysis corrects for stratification in genome-wide association studies. Nat Genet. 2006;38(8): 904–9. 16862161 10.1038/ng1847

[24] Kosoy R, Nassir R, Tian C, White PA, Butler LM, Silva G, et al Ancestry informative marker sets for determining continental origin and admixture proportions in common populations in America. Hum Mutat. 2009;30(1): 69–78. 10.1002/humu.20822 18683858 PMC3073397

[25] StataCorp. Stata Statistical Software: Release 12. College Station, TX: StataCorp LP; 2011.

[26] Law MH, Montgomery GW, Brown KM, Martin NG, Mann GJ, Hayward NK, et al Meta-analysis combining new and existing data sets confirms that the TERT-CLPTM1L locus influences melanoma risk. J Invest Dermatol. 2012;132(2): 485–7. 10.1038/jid.2011.322 21993562 PMC3258346

[27] Stacey SN, Sulem P, Masson G, Gudjonsson SA, Thorleifsson G, Jakobsdottir M, et al New common variants affecting susceptibility to basal cell carcinoma. Nat Genet. 2009;41(8): 909–14. 10.1038/ng.412 19578363 PMC2973331

[28] Wang Y, Broderick P, Webb E, Wu X, Vijayakrishnan J, Matakidou A, et al Common 5p15.33 and 6p21.33 variants influence lung cancer risk. Nat Genet. 2008;40(12): 1407–9. 10.1038/ng.273 18978787 PMC2695928

[29] Petersen GM, Amundadottir L, Fuchs CS, Kraft P, Stolzenberg-Solomon RZ, Jacobs KB, et al A genome-wide association study identifies pancreatic cancer susceptibility loci on chromosomes 13q22.1, 1q32.1 and 5p15.33. Nat Genet. 2010;42(3): 224–8. 10.1038/ng.522 20101243 PMC2853179

[30] Broderick P, Wang Y, Vijayakrishnan J, Matakidou A, Spitz MR, Eisen T, et al Deciphering the impact of common genetic variation on lung cancer risk: a genome-wide association study. Cancer Res. 2009;69(16): 6633–41. 10.1158/0008-5472.CAN-09-0680 19654303 PMC2754318

[31] Zheng SL, Stevens VL, Wiklund F, Isaacs SD, Sun J, Smith S, et al Two independent prostate cancer risk-associated Loci at 11q13. Cancer Epidemiol Biomarkers Prev. 2009;18(6): 1815–20. 10.1158/1055-9965.EPI-08-0983 19505914 PMC2802212

[32] Calcraft PJ, Ruas M, Pan Z, Cheng X, Arredouani A, Hao X, et al NAADP mobilizes calcium from acidic organelles through two-pore channels. Nature. 2009;459(7246): 596–600. 10.1038/nature08030 19387438 PMC2761823

[33] Kondo T, Hearing VJ. Update on the regulation of mammalian melanocyte function and skin pigmentation. Expert Rev Dermatol. 2011;6(1): 97–108. 21572549 10.1586/edm.10.70 PMC3093193

[34] Sulem P, Gudbjartsson DF, Stacey SN, Helgason A, Rafnar T, Jakobsdottir M, et al Two newly identified genetic determinants of pigmentation in Europeans. Nat Genet. 2008;40(7): 835–7. 10.1038/ng.160 18488028

[35] Gudbjartsson DF, Sulem P, Stacey SN, Goldstein AM, Rafnar T, Sigurgeirsson B, et al ASIP and TYR pigmentation variants associate with cutaneous melanoma and basal cell carcinoma. Nat Genet. 2008;40(7): 886–91. 10.1038/ng.161 18488027

[36] Tao S, Wang Z, Feng J, Hsu FC, Jin G, Kim ST, et al A genome-wide search for loci interacting with known prostate cancer risk-associated genetic variants. Carcinogenesis. 2012;33(3): 598–603. 10.1093/carcin/bgr316 22219177 PMC3291863

[37] Chung CC, Ciampa J, Yeager M, Jacobs KB, Berndt SI, Hayes RB, et al Fine mapping of a region of chromosome 11q13 reveals multiple independent loci associated with risk of prostate cancer. Hum Mol Genet. 2011;20(14): 2869–78. 10.1093/hmg/ddr189 21531787 PMC3118760

[38] Sturm RA. Molecular genetics of human pigmentation diversity. Hum Mol Genet. 2009;18(R1): R9–17. 10.1093/hmg/ddp003 19297406

[39] Scherer D, Kumar R. Genetics of pigmentation in skin cancer—a review. Mutat Res. 2010;705(2): 141–53. 10.1016/j.mrrev.2010.06.002 20601102

[40] Kim NH, Cheong KA, Lee TR, Lee AY. PDZK1 upregulation in estrogen-related hyperpigmentation in melasma. J Invest Dermatol. 2012;132(11): 2622–31. 10.1038/jid.2012.175 22696060

[41] Tadokoro T, Rouzaud F, Itami S, Hearing VJ, Yoshikawa K. The inhibitory effect of androgen and sex-hormone-binding globulin on the intracellular cAMP level and tyrosinase activity of normal human melanocytes. Pigment Cell Res. 2003;16(3): 190–7. 12753385 10.1034/j.1600-0749.2003.00019.x

[42] Randall JC, Winkler TW, Kutalik Z, Berndt SI, Jackson AU, Monda KL, et al Sex-stratified genome-wide association studies including 270,000 individuals show sexual dimorphism in genetic loci for anthropometric traits. PLoS Genet. 2013;9(6): e1003500 10.1371/journal.pgen.1003500 23754948 PMC3674993

