# Supplementary material for: Pleiotropic and Sex-Specific Effects of Cancer GWAS SNPs on Melanoma Risk in the Population Architecture Using Genomics and Epidemiology (PAGE) Study
Source: PLoS One. 2015 Mar 19;10(3):e0120491. doi: 10.1371/journal.pone.0120491 (PMC4366224; doi:10.1371/journal.pone.0120491)
Supplement: S1 File — Detailed descriptions for each of the five studies contributing to this analysis. (DOCX) [file pone.0120491.s001.docx]

Kocarnik et al.

Pleiotropic and sex-specific effects of cancer GWAS SNPs on melanoma risk in the Population Architecture using Genomics and Epidemiology (PAGE) study

**Supplementary Material –** Overview of case and control definition, as well as matching criteria, utilized by the five studies participating in this analysis. Additional information on each of the five participating studies is provided below.

| **Case definition** | |  |
| --- | --- | --- |
|  | EAGLE-BioVU | ≥3 mentions of ICD-9 codes 179.* (malignant melanoma of the skin) OR tumor registry entry for melanoma. |
|  | HPFS | Incident skin cancer from baseline to 2008 follow-up cycle, from the subcohort who gave a blood specimen in 1993-94. |
|  | MEC | Melanoma cases identified through Hawaii and Los Angeles SEER programs, in the subcohort with blood samples. |
|  | NHS | Incident skin cancer from baseline to 2008 follow-up cycle, from the subcohort who gave a blood specimen in 1989-90. |
|  | WHI | Incident melanoma through 2009, no previous cancer (except non-melanoma skin cancer) |
|  |  |  |
| **Control definition** | | |
|  | EAGLE-BioVU | No code for neoplasms, no ICD-9 codes between 140._ and 239._, no tumor registry entry or cancer related keywords in the problem list. |
|  | HPFS | Participants without a melanoma diagnosis, from the subcohort who gave a blood specimen in 1993-94. |
|  | MEC | Participants without melanoma history, preferentially selected from participants with biomarker data available. |
|  | NHS | Participants without a melanoma diagnosis, from the subcohort who gave a blood specimen in 1989-90. |
|  | WHI | Free of prevalent cancer (including non-melanoma skin cancer) and incident cancer (excluding non-melanoma skin cancer); |
|  |  | Additional controls: have either IGF-1 or IGFBP3, or have SHBG and ≥4 hormone measurements. |
|  |  |  |
| **Controls** | |  |
|  | EAGLE-BioVU | Matched and unmatched |
|  | HPFS | Unmatched |
|  | MEC | Matched and unmatched |
|  | NHS | Unmatched |
|  | WHI | Matched and unmatched |
|  |  |  |
| **Matching criteria** | | |
|  | EAGLE-BioVU | Sex, race/ethnicity, and age (± 5 years) |
|  | MEC | Sex, year of birth (± 1 year), ethnicity, date of blood draw (± 6 months), time of day of blood draw (AM or PM), fasting status (0.6, 6-8, 8-10, or 10+ hours), study site, alive at case diagnosis, and type of urin collection (first morning, overnight, none); |
|  |  | Secondary, relaxed matching on date of blood draw (± 1 year) and year of birth (± 5 years) |
|  | WHI | Age (± 1 year), enrollment date (± 1 year), race/ethnicity, randomization arms (OS flag, HRT assignments, CaD assignments), in prioritized cancer order. |

**Epidemiologic Architecture for Genes Linked to Environment (EAGLE) accessing BioVU (1)**. BioVU is a biorepository of DNA samples extracted from blood drawn for routine clinical care. DNA samples are linked to a de-identified version of the patient’s electronic medical records for research purposes unless the patient opts-out out of the biorepository via the consent to treatment form. The Vanderbilt electronic medical record (EMR) began accumulating clinical data in the early 1990s. Biological sample collection for BioVU began in 2007, with an accrual rate of ~700 samples per week. Updating of EMR information and genotyping of additional samples is ongoing. Genotyping was conducted by the Vanderbilt DNA Resources Core with the use of the mid-throughput Sequenom genotyping platform and TaqMan assays and the ABI Prism 7900HT Sequence Detection System (Applied Biosystems). Eight major cancers, including melanoma, were defined using tumor registry entries, billing codes (ICD9 codes), and procedure codes, as previously described (2). Two cancer-free controls matched by sex and race/ethnicity and frequency matched by age (roughly ± five years) were identified per cancer case. For melanoma, incident cases included those that were diagnosed up to 5 years before entrance into the study. When appropriate, group controls that were matched to cases of other cancers are used as additional controls in cancer-specific analyses to improve power.

**Multiethnic Cohort Study (MEC) (3)**. MEC was initiated in 1993 to investigate the impact of dietary and environmental factors on major chronic diseases, particularly cancer, in ethnically diverse populations in Hawai’i and California. The study recruited 96,810 men and 118,441 women aged 45 to 75 years between 1993 and 1996. Melanoma cases are identified through the Rapid Reporting System of the Hawai’i Tumor Registry and through quarterly linkage to the Los Angeles County Cancer Surveillance Program. Both registries are members of SEER. Eligible cases were incident cases with invasive melanoma occurring in the sub-cohort with blood sample (n~70,000). Controls were matched on sex, year of birth (± 1 year), ethnicity (White, African American, Hawaiian, Japanese, Latino), date of blood draw (± 6 months), time of day of blood draw (AM or PM), fasting status (0-6, 6-8, 8-10, or 10+ hours), study site, alive at case diagnosis, and type of urine (first morning, overnight, none). If no controls matched, then a relaxed criteria was used: date of blood draw (± 1 year), year of birth (± 5 years). Controls were preferentially selected from those participants with biomarker data available, such as lipids and glucose. A case-control set was created for each of the cancer types investigated as part of collaboration with PAGE (breast cancer, endometrial cancer, lung cancer, ovarian cancer, melanoma, Non-Hodgkin lymphoma, prostate cancer, and colorectal cancer). Where eligible, controls from case-control analyses of other cancers (for example, colorectal cancer) were added to the control set for the cancer of interest (here melanoma) to improve power. If results differ between analyses including and excluding these additional controls, only the results using the matched controls are used.

**Women’s Health Initiative (WHI)** **(5).** The Women’s Health Initiative (WHI) is a long-term health study of 161,808 post-menopausal women aged 50 to 79 years at 40 clinical centers throughout the United States. WHI comprises a Clinical Trial (CT) arm, an Observational Study (OS) arm, and several extension studies. The details of WHI have been previously described (5-6), and are available online (<https://cleo.whi.org/SitePages/Home.aspx>). As part of its participation in the PAGE project, cases and controls for various cancers (melanoma, non-Hodgkin’s lymphoma, and breast, lung, endometrial, ovarian, and colorectal cancer,) were selected in parallel for nested case-control analyses. For each cancer type, cases occurred through August 2009, and were excluded if they had baseline history of that cancer or a previous incident cancer. Participants with two or more incident cancers were only included as a case for the first cancer type. Controls were required to be free of prevalent caner (including non-melanoma skin cancer) and incident cancer (excluding non-melanoma skin cancer). Controls were matched to cases on age (± 1 year for Whites, ± 2 years for Blacks, ± 3 years for other race/ethnicities), enrollment date (± 365 days), race/ethnicity (White, Black, Hispanic, American Indian, Asian/Pacific Islander), and randomization arms (OS flag, HRT assignments, CaD assignments). Matching was done individually for each cancer type in sequential order: invasive breast cancer was matched first, followed by endometrial cancer, lung cancer, ovarian cancer, melanoma, Non-Hodgkin lymphoma, and colorectal cancer. After each outcome match, those controls selected were reused for the next outcome as the top priority to match from. Control selection was done in a time-forward manner. Each matching factor was given the same weight. Cases and controls are matched 1:1. The matching algorithm was allowed to select the closest match based on a criterion to minimize an overall distance measure (7). SAS code was available to implement this matching scheme. In order to increase sample sizes for cancer-specific analyses, controls from case-control analyses of other cancers (for example, colorectal cancer) were also used as additional controls for a cancer of interest (here melanoma) if a sensitivity analysis demonstrated no difference when doing so. An additional control set was also generated at the same time for a WHI analysis of hormones. For these hormone controls, participants were selected if they had either IGF-I or IGFBP3, or if they had SHBG and ≥4 hormones out of estrone, progesterone, free testosterone, bioavailable testosterone, free estradiol, or bioavailable estradiol. To improve power, these controls were also utilized as additional controls for cancer-specific case-control analyses, provided sensitivity analyses showed no difference when included.

**Nurses’ Health Study (NHS) (8-9).** The Nurses’ Health Study (NHS) was established in 1976, when 121,700 female registered nurses between the ages of 30 and 55 residing in 11 large US states completed and returned the initial self-administered questionnaire on their medical histories and baseline health-related exposures, forming the basis for the NHS cohort. Updated information has been obtained by questionnaires every 2 years, including exposure information on risk factors and outcome data with appropriate follow-up of reported disease events. Overall follow-up has been very high, with ~90% of participants continuing to complete questionnaires, even after more than 20 years. Information on melanoma development was first collected in the 1984 questionnaire. Eligible cases in this study consisted of women with incident skin cancer from the subcohort who gave a blood specimen in 1989–1990 (n = 32,826), and who had melanoma diagnosed any time after baseline up to the 2008 follow-up cycle. Eligible controls were participants from this subcohort without a melanoma diagnosis. All subjects were drawn from among the US non-Hispanic Caucasian women in this study. Continued below.

**Health Professionals Follow-up Study (HPFS) (10)**. The HPFS is a parallel prospective study to the Nurses’ Health Study (NHS). The HPFS cohort comprises 51,529 men who, in 1986, responded to a mailed questionnaire. The participants are U.S. male dentists, optometrists, osteopaths, podiatrists, pharmacists, and veterinarians born between 1910 and 1946. Biennial questionnaires collect disease and health-related information. Follow-up has been excellent, with 94% of the men responding to date. Information on melanoma development was first collected in the 1986 questionnaire. Between 1993 and 1994, 18,159 study participants provided blood samples by overnight courier. Eligible cases in this study consisted of men with incident skin cancer from the subcohort who gave a blood specimen, and who had melanoma diagnosed any time after baseline up to the 2008 follow-up cycle. Eligible controls were participants from this subcohort without a melanoma diagnosis. All subjects were drawn from among the US non-Hispanic Caucasian men in this study. Continued below.

**NHS and HPFS:** Both cohorts have conducted nested case-control studies of melanoma (11). To do so, both cohorts utilized data from previously conducted GWASs on different disease outcomes (NHS: breast cancer, coronary heart disease, type 2 diabetes, kidney stone, pancreatic cancer, and glaucoma; HPFS: coronary heart disease, type 2 diabetes, kidney stone, advanced prostate cancer, and glaucoma). For the purposes of conducting a melanoma GWAS, the controls from each of these prior GWAS were compiled (except for the kidney stone GWAS, in which both cases and controls were used). From this data, participants with melanoma diagnosis were classified as cases and participants without melanoma diagnosis were the controls. Added to this were any melanoma cases in both NHS and HPFS who had not been included in these previous GWAS. Genotyping of cases and controls occurred in the previous GWAS studies. For the breast cancer GWAS, genotyping in NHS was performed on the Illumina HumanHap550 array, as part of the National Cancer Institute’s Cancer Genetic Markers of Susceptibility (CGEMS) Project. For the coronary heart disease and type 2 diabetes GWASs, genotyping was performed using the Affymetrix 6.0 array. For the glaucoma GWAS, genotyping was performed using the Illumina HumanHap660 array. For the kidney stone, advanced prostate cancer, and melanoma GWASs, genotyping was performed using the Illumina HumanHap610 array. Based on the genotyped SNPs and haplotype information in the NCBI build 35 of phase II Hapmap CEU data, genotypes were imputed for >2.5 million SNPs using the program MACH (12). Only SNPs with imputation quality R^2^ > 0.95 in each study were included in the final analysis. A total of 1,579,307 SNPs were included in the final melanoma meta-analysis of the NHS and HPFS. Betas from each study were combined in a meta-analysis with weights proportional to the inverse variance of the beta in each study.

1. Roden DM, Pulley JM, Basford MA, et al. Development of a Large-Scale De-Identified DNA Biobank to Enable Personalized Medicine. *Clin Pharmacol Ther*. 2008;84(3):362-369.

2. Bush W, Boston J, Pendergrass SA, et al. Enabling high-throughput genotype-phenotype associations in the Epidemiologic Architecture for Genes Linked to Environment (EAGLE) project as part of the Population Architecture using Genomics and Epidemiology (PAGE) study. *Pacific Symposium on Biocomputing* 2013.

3. Kolonel LN, Henderson BE, Hankin JH, et al. A multiethnic cohort in Hawaii and Los Angeles: baseline characteristics. *Am J Epidemiol*. 2000;151(4):346-357.

5. Hays J, Hunt JR, Hubbell FA, et al. The women's health initiative recruitment methods and results. *Annals of Epidemiology*. 2003;13(9, Supplement):S18-S77.

6. Design of the Women's Health Initiative Clinical Trial and Observational Study. *Control Clin Trials*. 1998;19(1):61-109.

7. Bergstralh EJ & Kosanke JL. Computerized matching of cases to controls. Technical Report #56, Department of Health Sciences Research, Mayo Clinic, Rochester MN. April 1995.

8. Colditz GA & Hanksinson SE. The Nurses’ Health Study: Lifestyle and health among women. *Nat Rev Cancer*. 2005;5:388-96.

9. Belanger CF, Hennekens CH, Rosner B*, et al.* The nurses' health study. *Am J Nurs* 1978;78(6):1039-40.

10. Rimm EB, Stampfer MJ, Colditz GA*, et al.* Validity of self-reported waist and hip circumferences in men and women. *Epidemiology* 1990;1(6):466-73.

11. Meng S, Song F, Chen H, *et al.* No assocaition between Parkinson Disease alleles and the risk of melanoma. *Cancer Epidemiol Biomarkers Prev* 2012;12(1):243-5.

12. Li Y, Willer CJ, Ding J, Scheet P and Abecasis GR (2010) MaCH: using sequence and genotype data to estimate haplotypes and unobserved genotypes. *Genet Epidemiol* 34:816-834.
