# Supplementary material for: Pleiotropic and Sex-Specific Effects of Cancer GWAS SNPs on Melanoma Risk in the Population Architecture Using Genomics and Epidemiology (PAGE) Study
Source: PLoS One. 2015 Mar 19;10(3):e0120491. doi: 10.1371/journal.pone.0120491 (PMC4366224; doi:10.1371/journal.pone.0120491)
Supplement: S1 Table — Provides the allele associated with increased risk in the original cancer GWAS publication, as well as the chromosomal location of the SNP and gene. (DOCX) [file pone.0120491.s002.docx]

Kocarnik et al.

Evaluation of pleiotropic and sex-specific effects of cancer GWAS SNPs on melanoma risk in the Population Architectures using Genomics and Epidemiology (PAGE) study

| **Table S1 - Full list of 181 SNPs evaluated for an association with melanoma.** Provides the allele associated with increased risk in the original cancer GWAS publication, as well as the chromosomal location of the SNP and gene. | | | | | | | |
| --- | --- | --- | --- | --- | --- | --- | --- |
|  |  |  |  |  |  |  |  |
| SNP | Risk allele | Original cancer association | | Mapped Gene | Region | Position | Author Year Journal |
|  |  |  |  |  |  |  |  |
| rs10086908 | T | Prostate cancer | | *Intergenic* | 8q24.21 | 128081119 | Al Olama 2009 Nat Genet |
| rs10090154 | A | Prostate cancer | | *Intergenic* | 8q24.21 | 128601319 | Al Olama 2009 Nat Genet |
| rs1016343 | T | Prostate cancer | | *Intergenic* | 8q24.21 | 128162479 | Eeles 2008 Nat Genet |
| rs10220831 | T | Non-Hodgkin lymphoma (CLL) | | *Intergenic* | 15q23 | 67779168 | Di Bernardo 2008 NatGenet |
| rs10263639 | C | Breast cancer | | *Intergenic* | 7q11.22 | 66696702 | Murabito 2007 BMC Med Genet |
| rs1036935 | T | Non-Hodgkin lymphoma (CLL) | | *Intergenic* | 18q21.1 | 46097532 | Crowther-Swanepoel 2010 Nat Genet |
| rs10411210 | C | Colorectal cancer | | *RHPN2* | 19q13.11 | 38224140 | Houlston 2008 Nat Genet |
| rs1045485 | G | Breast cancer | | *CASP8* | 2q33.1 | 201857834 | Cox 2007 Nat Genet |
| rs10464870 | C | Glioma |  | *Intergenic* | 8q24.21 | 130547005 | Shete 2009 Nat Genet |
| rs10483813 | T | Breast cancer | | *RAD51L1* | 14q24.1 | 68101037 | Thomas 2009 Nat Genet |
| rs10486567 | G | Prostate cancer | | *JAZF1* | 7p15.2 | 27943088 | Eeles 2008 Nat Genet |
| rs10490113 | C | Breast cancer | | *Intergenic* | 2p16.1 | 59352851 | Murabito 2007 BMC Med Genet |
| rs10505477 | A | Colorectal cancer | | *Intergenic* | 8q24.21 | 128476625 | Zanke 2007 Nat Genet |
| rs1051730 | A | Lung cancer | | *CHRNA3* | 15q25.1 | 76681394 | Landi 2009 Am J Hum Genet |
| rs10778826 | A | Prostate cancer | | *PPFIA2* | 12q21.31 | 80626985 | Eeles 2009 Nat Genet |
| rs10795668 | G | Colorectal cancer | | *Intergenic* | 10p14 | 8741255 | Tomlinson 2008 Nat Genet |
| rs10821936 | C | Leukemia (ALL) | | *ARID5B* | 10q21.2 | 63393583 | Trevino 2009 Nat Genet |
| rs10896449 | G | Prostate cancer | | *Intergenic* | 11q13.3 | 68751243 | Thomas 2008 Nat Genet |
| rs10941679 | G | Breast cancer | | *Intergenic* | 5p12 | 44742255 | Stacey 2007 Nat Genet |
| rs10974944 | G | Leukemia (Myeloid) | | *JAK2* | 9p24.1 | 5060831 | Kilpivaara 2009 Nat Genet |
| rs10993994 | T | Prostate cancer | | *Intergenic* | 10q11.23 | 51219502 | Eeles 2008 Nat Genet |
| SNP | Risk allele | Original cancer association | | Mapped Gene | Region | Position | Author Year Journal |
| rs10994982 | A | Leukemia (ALL) | | *ARID5B* | 10q21.2 | 63380110 | Trevino 2009 Nat Genet |
| rs11083846 | A | Non-Hodgkin lymphoma (CLL) | | *PRKD2* | 19q13.32 | 51899494 | Di Bernardo 2008 NatGenet |
| rs11155133 | G | Leukemia (ALL) | | *Intergenic* | 6q24.1 | 141211518 | Trevino 2009 Nat Genet |
| rs11170164 | A | Basal cell carcinoma | | *KRT5* | 12q13.13 | 51199935 | Stacey 2009 Nat Genet |
| rs11228565 | A | Prostate cancer | | *Intergenic* | 11q13.3 | 68735156 | Gudmundsson 2009 Nat Genet |
| rs11249433 | C | Breast cancer | | *EMBP1* | 1p11.2 | 120982136 | Thomas 2009 Nat Genet |
| rs11649338 | C | Breast cancer | | *Intergenic* | 16q23.1 | 73028921 | Kibriya 2009 Breast Cancer Res Treat |
| rs11649743 | G | Prostate cancer | | *HNF1B* | 17q12 | 33149092 | Sun 2008 Nat Genet |
| rs11668878 | T | Non-Hodgkin lymphoma (CLL) | | *Intergenic* | 19q13.32 | 51960213 | Crowther-Swanepoel 2010 Nat Genet |
| rs11861609 | C | Prostate cancer | | *CDH13* | 16q23.3 | 81942167 | Eeles 2009 Nat Genet |
| rs12155172 | A | Prostate cancer | | *Intergenic* | 7p15.3 | 20961016 | Eeles 2009 Nat Genet |
| rs1219648 | G | Breast cancer | | *FGFR2* | 10q26.13 | 123336180 | Hunter 2007 Nat Genet |
| rs1229984 | C | Esophageal cancer | | *ADH1B* | 4q23 | 100458342 | McKay 2011 PLoS Genet |
| rs12418451 | A | Prostate cancer | | *Intergenic* | 11q13.3 | 68691995 | Hsu 2009 Cancer Res |
| rs12500426 | A | Prostate cancer | | *PDLIM5* | 4q22.3 | 95733632 | Eeles 2009 Nat Genet |
| rs12543663 | C | Prostate cancer | | *Intergenic* | 8q24.21 | 127993841 | Al Olama 2009 Nat Genet |
| rs12621278 | A | Prostate cancer | | *ITGA6* | 2q31.1 | 173019799 | Eeles 2009 Nat Genet |
| rs13252298 | A | Prostate cancer | | *Intergenic* | 8q24.21 | 128164338 | Al Olama 2009 Nat Genet |
| rs13254738 | C | Prostate cancer | | *Intergenic* | 8q24.21 | 128173525 | Haiman 2007 Nat Gent |
| rs13281615 | T | Breast cancer | | *Intergenic* | 8q24.21 | 128424800 | Easton 2007 Nature |
| rs13387042 | A | Breast cancer | | *Intergenic* | 2q35 | 217614077 | Thomas 2009 Nat Genet |
| rs13397985 | G | Non-Hodgkin lymphoma (CLL) | | *SP140* | 2q37.1 | 230799467 | Di Bernardo 2008 NatGenet |
| rs1412829 | C | Glioma (high-grade) | | *CDKN2BAS1* | 9p21.3 | 22033926 | Wrensch 2009 Nat Genet |
| rs1447295 | A | Prostate cancer | | *Intergenic* | 8q24.21 | 128554220 | Gudmundsson 2007 Nat Genet |
| rs1465618 | T | Prostate cancer | | *THADA* | 2p21 | 43407453 | Eeles 2009 Nat Genet |
| rs1512268 | T | Prostate cancer | | *Intergenic* | 8p21.2 | 23582408 | Eeles 2009 Nat Genet |
| rs1530057 | A | Lung cancer | | *RBMS3* | 3p24.1 | 29550467 | Broderick 2009 Cancer Res |
| rs1571801 | T | Prostate cancer | | *DAB2IP* | 9q33.2 | 123467194 | Dugan 2007 J Natl Cancer Inst |
| rs157935 | T | Basal cell carcinoma | | *Intergenic* | 7q32.3 | 130236093 | Stacey 2009 Nat Genet |
| rs167020 | A | Pancreatic cancer | | *Intergenic* | 7q36.3 | 155312494 | Amundadottir 2009 Nat Genet |
| SNP | Risk allele | Original cancer association | | Mapped Gene | Region | Position | Author Year Journal |
| rs16886165 | G | Breast cancer | | *Intergenic* | 5q11.2 | 56058840 | Thomas 2009 Nat Genet |
| rs16892766 | C | Colorectal cancer | | *Intergenic* | 8q23.3 | 117699864 | Tomlinson 2008 Nat Genet |
| rs16901979 | A | Prostate cancer | | *Intergenic* | 8q24.21 | 128194098 | Gudmundsson 2009 Nat Genet |
| rs16902094 | G | Prostate cancer | | *Intergenic* | 8q24.21 | 128389528 | Gudmundsson 2009 Nat Genet |
| rs17021918 | C | Prostate cancer | | *PDLIM5* | 4q22.3 | 95781900 | Eeles 2009 Nat Genet |
| rs172310 | A | Pancreatic cancer | | *Intergenic* | 7q36.3 | 155308388 | Amundadottir 2009 Nat Genet |
| rs17483466 | G | Non-Hodgkin lymphoma (CLL) | | *ACOXL* | 2q13 | 111513929 | Di Bernardo 2008 NatGenet |
| rs1859962 | G | Prostate cancer | | *Intergenic* | 17q24.3 | 66620348 | Eeles 2008 Nat Genet |
| rs1876206 | G | Breast cancer | | *FBN1* | 15q21.1 | 46687878 | Murabito 2007 BMC Med Genet |
| rs189897 | A | Nasopharyngeal carcinoma | | *ITGA9* | 3p22.2 | 37493549 | Ng 2009 J Hum Genet |
| rs1926203 | T | Lung cancer | | *ACTA2* | 10q23.31 | 90717314 | Broderick 2009 Cancer Res |
| rs1926657 | T | Breast cancer | | *ABCC4* | 13q32.1 | 94672957 | Murabito 2007 BMC Med Genet |
| rs1978503 | G | Breast cancer | | *Intergenic* | 18q21.2 | 51815280 | Murabito 2007 BMC Med Genet |
| rs2046210 | A | Breast cancer | | *Intergenic* | 6q25.1 | 151990059 | Zheng 2009 Nat Genet |
| rs2075555 | T | Breast cancer | | *COL1A1* | 17q21.33 | 45629290 | Murabito 2007 BMC Med Genet |
| rs2089222 | A | Leukemia (ALL) | | *MAP1LC3B2* | 12q24.22 | 115487041 | Trevino 2009 Nat Genet |
| rs210138 | G | Testicular germ cell tumor | | *BAK1* | 6p21.31 | 33650516 | Rapley 2009 Nat Genet |
| rs2151280 | C | Basal cell carcinoma | | *CDNK2BAS1* | 9p21.3 | 22024719 | Stacey 2009 Nat Genet |
| rs2167364 | C | Leukemia (ALL) | | *DDC* | 7p12.1 | 50533321 | Trevino 2009 Nat Genet |
| rs2180341 | G | Breast cancer | | *RNF146* | 6q22.33 | 127642323 | Gold 2008 Proc Natl Acad Sci |
| rs2191566 | G | Leukemia (ALL) | | *ZNF230* | 19q13.31 | 49203229 | Trevino 2009 Nat Genet |
| rs2239633 | G | Leukemia (ALL) | | *Intergenic* | 14q11.2 | 22658897 | Papaemmanuil 2009 Nat Genet |
| rs2242041 | G | Leukemia (ALL) | | *DDC* | 7p12.1 | 50496943 | Trevino 2009 Nat Genet |
| rs2294008 | T | Bladder cancer | | *PSCA* | 8q24.3 | 143758933 | Rothman 2010 Nat Genet |
| rs2456449 | G | Non-Hodgkin lymphoma (CLL) | | *Intergenic* | 8q24.21 | 128262163 | Crowther-Swanepoel 2010 Nat Genet |
| rs2660753 | T | Prostate cancer | | *Intergenic* | 3p12.1 | 87193364 | Eeles 2008 Nat Genet |
| rs266849 | A | Prostate cancer | | *Intergenic* | 19q13.33 | 56040902 | Eeles 2008 Nat Genet |
| rs2710647 | C | Prostate cancer | | *EHBP1* | 2p15 | 63067474 | Eeles 2009 Nat Genet |
| rs2735839 | G | Prostate cancer | | *Intergenic* | 19q13.33 | 56056435 | Eeles 2008 Nat Genet |
| rs2736100 | G | Glioma |  | *TERT* | 5p15.33 | 1339516 | Shete 2009 Nat Genet |
| SNP | Risk allele | Original cancer association | | Mapped Gene | Region | Position | Author Year Journal |
| rs2808630 | C | Lung cancer | | *Intergenic* | 1q23.2 | 157947492 | Amos 2008 Nat Genet |
| rs2853676 | A | Glioma |  | *TERT* | 5p15.33 | 1341547 | Shete 2009 Nat Genet |
| rs2928679 | A | Prostate cancer | | *Intergenic* | 8p21.2 | 23494920 | Thomas 2008 Nat Genet |
| rs2981578 | G | Breast cancer | | *FGFR2* | 10q26.13 | 123330301 | Turnbull 2010 Nat Genet |
| rs2981579 | T | Breast cancer | | *FGFR2* | 10q26.13 | 123327325 | Thomas 2009 Nat Genet |
| rs2981582 | T | Breast cancer | | *FGFR2* | 10q26.13 | 123342307 | Easton 2007 Nature |
| rs305061 | T | Non-Hodgkin lymphoma (CLL) | | *Intergenic* | 16q24.1 | 84533160 | Crowther-Swanepoel 2010 Nat Genet |
| rs3117582 | C | Lung cancer | | *Intergenic* | 6p21.33 | 31728499 | Landi 2009 Am J Hum Genet |
| rs3131379 | T | Lung cancer | | *MSH5* | 6p21.33 | 31829012 | Harley 2008 Nat Genet |
| rs31489 | C | Lung cancer | | *CLPTM1L* | 5p15.33 | 1395714 | Landi 2009 Am J Hum Genet |
| rs3750817 | C | Breast cancer | | *FGFR2* | 10q26.13 | 123322567 | Prentice 2009 Cancer Epi Biomarkers Prev |
| rs3790844 | T | Pancreatic cancer | | *NR5A2* | 1q32.1 | 198274055 | Petersen 2010 Nat Genet |
| rs3802842 | C | Colorectal cancer | | *C11orf93* | 11q23.1 | 110676919 | Tenesa 2008 Nat Genet |
| rs3803662 | T | Breast cancer | | *Intergenic* | 16q12.1 | 51143842 | Thomas 2009 Nat Genet |
| rs3814113 | T | Ovarian cancer | | *Intergenic* | 9p22.2 | 16905021 | Song 2009 Nat Genet |
| rs3817198 | C | Breast cancer | | *LSP1* | 11p15.5 | 1865582 | Easton 2007 Nature |
| rs401681 | C | Lung cancer | | *CLPTM1L* | 5p15.33 | 1375087 | Wang 2008 Nat Genet |
| rs402710 | C | Lung cancer | | *CLPTM1L* | 5p15.33 | 1373722 | McKay 2008 Nat Genet |
| rs4132601 | C | Leukemia (ALL) | | *IKZF1* | 7p12.2 | 50438098 | Papaemmanuil 2009 Nat Genet |
| rs4242382 | A | Prostate cancer | | *Intergenic* | 8q24.21 | 128586755 | Thomas 2008 Nat Genet |
| rs4254535 | C | Lung cancer | | *Intergenic* | 2p13.3 | 69051892 | Broderick 2009 Cancer Res |
| rs4295627 | G | Glioma |  | *Intergenic* | 8q24.21 | 130754639 | Shete 2009 Nat Genet |
| rs4324715 | T | Testicular cancer | | *Intergenic* | 5q31.3 | 141649691 | Kanetsky 2009 Nat Genet |
| rs4324798 | A | Lung cancer | | *Intergenic* | 6p22.1 | 28884096 | Landi 2009 Am J Hum Genet |
| rs4415084 | T | Breast cancer | | *Intergenic* | 5p12 | 44698272 | Stacey 2007 Nat Genet |
| rs4430796 | G | Prostate cancer | | *HNF1B* | 17q12 | 33172153 | Gudmundsson 2009 Nat Genet |
| rs4444235 | C | Colorectal cancer | | *Intergenic* | 14q22.2 | 53480669 | Houlston 2008 Nat Genet |
| rs445114 | T | Prostate cancer | | *Intergenic* | 8q24.21 | 128392363 | Gudmundsson 2009 Nat Genet |
| rs4474514 | A | Testicular cancer | | *KITLG* | 12q21.32 | 87478090 | Kanetsky 2009 Nat Genet |
| rs458685 | C | Breast cancer | | *GRIK1* | 21q21.3 | 30099382 | Murabito 2007 BMC Med Genet |
| SNP | Risk allele | Original cancer association | | Mapped Gene | Region | Position | Author Year Journal |
| rs4624820 | A | Testicular germ cell tumor | | *Intergenic* | 5q31.3 | 141661972 | Rapley 2009 Nat Genet |
| rs4657482 | A | Testicular germ cell tumor | | *UCK2* | 1q24.1 | 164098273 | Rapley 2009 Nat Genet |
| rs4699052 | C | Testicular germ cell tumor | | *Intergenic* | 4q24 | 104357239 | Rapley 2009 Nat Genet |
| rs4779584 | T | Colorectal cancer | | *Intergenic* | 15q13.3 | 30782048 | Tomlinson 2008 Nat Genet |
| rs4782780 | T | Prostate cancer | | *CDH13* | 16q23.3 | 81960548 | Eeles 2009 Nat Genet |
| rs4809324 | C | Glioma (high-grade) | | *RTEL1* | 20q13.33 | 61788664 | Wrensch 2009 Nat Genet |
| rs4857841 | A | Prostate cancer | | *EEFSEC* | 3q21.3 | 129529333 | Gudmundsson 2009 Nat Genet |
| rs4939827 | T | Colorectal cancer | | *SMAD7* | 18q21.1 | 44707461 | Tenesa 2008 Nat Genet |
| rs4961199 | A | Prostate cancer | | *Intergenic* | 8q21.3 | 87650060 | Eeles 2008 Nat Genet |
| rs4962416 | C | Prostate cancer | | *CTBP2* | 10q26.13 | 126686862 | Thomas 2008 Nat Genet |
| rs4973768 | T | Breast cancer | | *SLC4A7* | 3p24.1 | 27391017 | Ahmed 2009 Nat Genet |
| rs4975616 | A | Lung cancer | | *Intergenic* | 5p15.33 | 1368660 | Broderick 2009 Cancer Res |
| rs4977756 | G | Glioma |  | *CDKN2BAS1* | 9p21.3 | 22058652 | Shete 2009 Nat Genet |
| rs498872 | T | Glioma |  | *PHLDB1* | 11q23.3 | 117982577 | Shete 2009 Nat Genet |
| rs505922 | C | Pancreatic cancer | | *ABO* | 9q34.2 | 135139050 | Amundadottir 2009 Nat Genet |
| rs5759167 | G | Prostate cancer | | *Intergenic* | 22q13.2 | 41830156 | Eeles 2009 Nat Genet |
| rs5945572 | A | Prostate cancer | | *Intergenic* | Xp11.22 | 51246423 | Gudmundsson 2008 Nat Genet |
| rs5945619 | C | Prostate cancer | | *Intergenic* | Xp11.22 | 51258412 | Eeles 2008 Nat Genet |
| rs6001749 | G | Prostate cancer | | *TNRC6B* | 22q13.1 | 38805272 | Sun 2009 Cancer Res |
| rs6010620 | G | Glioma |  | *RTEL1* | 20q13.33 | 61780283 | Shete 2009 Nat Genet |
| rs620861 | G | Prostate cancer | | *Intergenic* | 8q24.21 | 128404855 | Eeles 2009 Nat Genet |
| rs630014 | C | Pancreatic cancer | | *ABO* | 9q34.2 | 135139543 | Amundadottir 2009 Nat Genet |
| rs6435862 | G | Neuroblastoma (high-risk) | | *BARD1* | 2q35 | 215380791 | Capasso 2009 Nat Genet |
| rs6457327 | C | NHL (Follicular lymphoma) | | *Intergenic* | 6p21.33 | 31182009 | Skibola 2009 Nat Genet |
| rs6465657 | C | Prostate cancer | | *LMTK2* | 7q21.3 | 97654263 | Eeles 2008 Nat Genet |
| rs6504950 | G | Breast cancer | | *STXBP4* | 17q22 | 50411470 | Ahmed 2009 Nat Genet |
| rs6556756 | G | Breast cancer | | *Intergenic* | 5q34 | 163821858 | Murabito 2007 BMC Med Genet |
| rs671 | A | Esophageal cancer | | *ALDH2* | 12q24.12 | 110726149 | Cui 2009 Gastroenterology |
| rs6939340 | G | Neuroblastoma (high-risk) | | *FLJ22536* | 6p22.3 | 22247983 | Maris 2008 N Engl J Med |
| rs6983267 | G | Colorectal cancer | | *Intergenic* | 8q24.21 | 128482487 | Tomlinson 2008 Nat Genet |
| SNP | Risk allele | Original cancer association | | Mapped Gene | Region | Position | Author Year Journal |
| rs6983561 | C | Prostate cancer | | *Intergenic* | 8q24.21 | 128176062 | Al Olama 2009 Nat Genet |
| rs7000448 | T | Prostate cancer | | *Intergenic* | 8q24.21 | 128510352 | Al Olama 2009 Nat Genet |
| rs7014346 | A | Colorectal cancer | | *Intergenic* | 8q24.21 | 128493974 | Tenesa 2008 Nat Genet |
| rs7089424 | C | Leukemia (ALL) | | *ARID5B* | 10q21.2 | 63422165 | Papaemmanuil 2009 Nat Genet |
| rs710521 | A | Urinary bladder cancer | | *Intergenic* | 3q28 | 191128627 | Rothman 2010 Nat Genet |
| rs7117034 | T | Prostate cancer | | *Intergenic* | 11q13.3 | 68731718 | Gudmundsson 2009 Nat Genet |
| rs7127900 | A | Prostate cancer | | *Intergenic* | 11p15.5 | 2190150 | Thomas 2008 Nat Genet |
| rs7176508 | A | Non-Hodgkin lymphoma (CLL) | | *Intergenic* | 15q23 | 67806044 | Di Bernardo 2008 NatGenet |
| rs719725 | A | Colorectal cancer | | *Intergenic* | 9p24 | 6355683 | Zanke 2007 Nat Genet |
| rs721048 | A | Prostate cancer | | *EHBP1* | 2p15 | 62985235 | Gudmundsson 2008 Nat Genet |
| rs735665 | A | Non-Hodgkin lymphoma (CLL) | | *Intergenic* | 11q24.1 | 122866607 | Conde 2010 Nat Genet |
| rs748404 | T | Lung cancer | | *Intergenic* | 15q15.2 | 41346523 | Broderick 2009 Cancer Res |
| rs7501939 | C | Prostate cancer | | *HNF1B* | 17q12 | 33175269 | Dugan 2007 J Natl Cancer Inst |
| rs7538876 | A | Basal cell carcinoma | | *PADI6* | 1p36.13 | 17594950 | Stacey 2008 Nat Genet |
| rs757978 | A | Non-Hodgkin lymphoma (CLL) | | *FARP2* | 2q37.3 | 242019774 | Crowther-Swanepoel 2010 Nat Genet |
| rs7626795 | G | Lung cancer | | *IL1RAP* | 3q28 | 191833155 | Amos 2008 Nat Genet |
| rs7679673 | C | Prostate cancer | | *Intergenic* | 4q24 | 106280983 | Eeles 2009 Nat Genet |
| rs7809758 | G | Leukemia (ALL) | | *DDC* | 7p12.1 | 50540827 | Papaemmanuil 2009 Nat Genet |
| rs7837688 | T | Prostate cancer | | *Intergenic* | 8q24.21 | 128608542 | Takata 2010 Nat Genet |
| rs7841060 | G | Prostate cancer | | *Intergenic* | 8q24.21 | 128165659 | Yeager 2009 Nat Genet |
| rs7931342 | G | Prostate cancer | | *Intergenic* | 11q13.3 | 68751073 | Eeles 2008 Nat Genet |
| rs801114 | G | Basal cell carcinoma | | *Intergenic* | 1q42.13 | 227064458 | Stacey 2008 Nat Genet |
| rs8034191 | C | Lung cancer | | *AGPHD1* | 15q25.1 | 76593078 | Broderick 2009 Cancer Res |
| rs8042374 | G | Lung cancer | | *CHRNA3* | 15q25.1 | 76695087 | Wang 2008 Nat Genet |
| rs8102476 | C | Prostate cancer | | *Intergenic* | 19q13.2 | 43427453 | Gudmundsson 2009 Nat Genet |
| rs872071 | G | Non-Hodgkin lymphoma (CLL) | | *IRF4* | 6p25.3 | 356064 | Di Bernardo 2008 NatGenet |
| rs889312 | C | Breast cancer | | *Intergenic* | 5q11.2 | 56067641 | Easton 2007 Nature |
| rs9295740 | A | Lung cancer | | *Intergenic* | 6p22.1 | 27797481 | Wang 2008 Nat Genet |
| rs931794 | G | Lung cancer | | *AGPHD1* | 15q25.1 | 76613235 | Amos 2008 Nat Genet |
| rs9364554 | T | Prostate cancer | | *SLC22A3* | 6q25.3 | 160753654 | Eeles 2008 Nat Genet |
| SNP | Risk allele | Original cancer association | | Mapped Gene | Region | Position | Author Year Journal |
| rs944289 | T | Thyroid cancer | | *Intergenic* | 14q13.3 | 35718997 | Gudmundsson 2009 Nat Genet |
| rs9543325 | C | Pancreatic cancer | | *Intergenic* | 13q22.1 | 72814629 | Petersen 2010 Nat Genet |
| rs961253 | A | Colorectal cancer | | *Intergenic* | 20p12.3 | 6352281 | Houlston 2008 Nat Genet |
| rs9623117 | C | Prostate cancer | | *Intergenic* | 22q13.1 | 38782065 | Sun 2009 Cancer Res |
| rs9642880 | T | Urinary bladder cancer | | *Intergenic* | 8q24.21 | 128787250 | Rothman 2010 Nat Genet |
| rs965513 | A | Thyroid cancer | | *Intergenic* | 9q22.33 | 99595930 | Gudmundsson 2009 Nat Genet |
| rs981782 | T | Breast cancer | | *HCN1* | 5p12 | 45321475 | Easton 2007 Nature |
| rs9929218 | G | Colorectal cancer | | *CDH1* | 16q22.1 | 67378447 | Houlston 2008 Nat Genet |
| rs995030 | G | Testicular germ cell tumor | | *KITLG* | 12q21.32 | 87414802 | Rapley 2009 Nat Genet |
| rs999737 | C | Breast cancer | | *RAD51B* | 14q24.1 | 68104435 | Thomas 2009 Nat Genet |
