# Supplementary material for: Pleiotropic and Sex-Specific Effects of Cancer GWAS SNPs on Melanoma Risk in the Population Architecture Using Genomics and Epidemiology (PAGE) Study
Source: PLoS One. 2015 Mar 19;10(3):e0120491. doi: 10.1371/journal.pone.0120491 (PMC4366224; doi:10.1371/journal.pone.0120491)
Supplement: S2 Table — Results for the association between melanoma and each of the 181 SNPs. (DOCX) [file pone.0120491.s003.docx]

Kocarnik et al.

Pleiotropic and sex-specific effects of cancer GWAS SNPs on melanoma risk in the Population Architecture using Genomics and Epidemiology (PAGE) study

| **Table S2 - Results for the association between melanoma and of each of the 181 SNPs.** SNPs are in numerical order, and P-values for the association with melanoma < 0.05 are in bold. | | | | | | | | | | | |  |
| --- | --- | --- | --- | --- | --- | --- | --- | --- | --- | --- | --- | --- |
| SNP | Coded allele | Previous trait association |  |  | Beta | SE | P-value | # studies | n | OR | 95% CI | Study P-heterogeneity |
| rs10086908 | T | Prostate cancer |  |  | -0.03 | 0.04 | 0.43 | 4 | 16,840 | 0.97 | (0.89 - 1.05) | 0.26 |
| rs10090154 | A | Prostate cancer |  |  | -0.04 | 0.09 | 0.62 | 2 | 10,605 | 0.96 | (0.81 - 1.13) | 0.39 |
| rs1016343 | T | Prostate cancer |  |  | 0.12 | 0.07 | 0.07 | 2 | 7,188 | 1.13 | (0.99 - 1.29) | 0.19 |
| rs10220831 | T | Non-Hodgkin lymphoma (CLL) | | | 0.04 | 0.06 | 0.55 | 3 | 7,987 | 1.04 | (0.92 - 1.17) | 0.63 |
| rs10263639 | C | Breast cancer |  |  | 0.02 | 0.06 | 0.79 | 3 | 11,392 | 1.02 | (0.9 - 1.15) | 0.12 |
| rs1036935 | T | Non-Hodgkin lymphoma (CLL) | | | 0.00 | 0.06 | 0.93 | 3 | 11,399 | 1 | (0.9 - 1.12) | 0.04 |
| rs10411210 | C | Colorectal cancer | |  | 0.13 | 0.07 | **0.046** | 3 | 15,971 | 1.14 | (1 - 1.3) | 0.68 |
| rs1045485 | G | Breast cancer |  |  | -0.10 | 0.06 | 0.08 | 5 | 21,018 | 0.91 | (0.81 - 1.01) | 0.03 |
| rs10464870 | C | Glioma |  |  | 0.01 | 0.07 | 0.86 | 2 | 7,208 | 1.01 | (0.89 - 1.16) | 0.24 |
| rs10483813 | T | Breast cancer |  |  | -0.02 | 0.07 | 0.78 | 3 | 8,057 | 0.98 | (0.86 - 1.12) | 0.10 |
| rs10486567 | G | Prostate cancer |  |  | 0.07 | 0.04 | 0.09 | 4 | 18,427 | 1.08 | (0.99 - 1.17) | 0.03 |
| rs10490113 | C | Breast cancer |  |  | 0.02 | 0.06 | 0.75 | 4 | 20,122 | 1.02 | (0.91 - 1.14) | 0.95 |
| rs10505477 | A | Colorectal cancer | |  | 0.03 | 0.05 | 0.55 | 3 | 13,144 | 1.03 | (0.94 - 1.12) | 0.84 |
| rs1051730 | A | Lung cancer |  |  | -0.01 | 0.04 | 0.76 | 5 | 22,127 | 0.99 | (0.92 - 1.06) | 0.34 |
| rs10778826 | A | Prostate cancer |  |  | 0.03 | 0.03 | 0.40 | 5 | 22,134 | 1.03 | (0.96 - 1.1) | 0.74 |
| rs10795668 | G | Colorectal cancer | |  | 0.02 | 0.04 | 0.56 | 5 | 22,021 | 1.02 | (0.95 - 1.1) | 0.33 |
| rs10821936 | C | Leukemia (ALL) |  |  | 0.03 | 0.04 | 0.45 | 4 | 13,333 | 1.03 | (0.95 - 1.13) | 0.92 |
| rs10896449 | G | Prostate cancer |  |  | 0.05 | 0.03 | 0.12 | 5 | 22,092 | 1.05 | (0.99 - 1.12) | 0.53 |
| rs10941679 | G | Breast cancer |  |  | -0.08 | 0.06 | 0.24 | 2 | 7,207 | 0.93 | (0.82 - 1.05) | 0.62 |
| rs10974944 | G | Leukemia (Myeloid) | | | -0.04 | 0.05 | 0.43 | 4 | 13,341 | 0.96 | (0.88 - 1.06) | 0.33 |
| rs10993994 | T | Prostate cancer |  |  | -0.01 | 0.04 | 0.84 | 3 | 16,015 | 0.99 | (0.92 - 1.07) | 0.02 |
| SNP | Coded allele | Previous trait association |  |  | Beta | SE | P-value | # studies | n | OR | 95% CI | Study P-heterogeneity |
| rs10994982 | A | Leukemia (ALL) |  |  | 0.05 | 0.04 | 0.23 | 4 | 13,307 | 1.05 | (0.97 - 1.15) | 0.61 |
| rs11083846 | A | Non-Hodgkin lymphoma (CLL) | | | -0.03 | 0.07 | 0.63 | 2 | 7,214 | 0.97 | (0.85 - 1.1) | 0.81 |
| rs11155133 | G | Leukemia (ALL) |  |  | -0.31 | 0.29 | 0.29 | 2 | 7,219 | 0.73 | (0.41 - 1.3) | 0.62 |
| rs11170164 | A | Basal cell carcinoma | |  | -0.10 | 0.23 | 0.66 | 1 | 1,925 | 0.91 | (0.58 - 1.41) | . |
| rs11228565 | A | Prostate cancer |  |  | 0.06 | 0.05 | 0.22 | 2 | 14,026 | 1.06 | (0.96 - 1.17) | 4.38E-04 |
| rs11249433 | C | Breast cancer |  |  | -0.01 | 0.04 | 0.88 | 3 | 15,612 | 0.99 | (0.92 - 1.08) | 0.53 |
| rs11649338 | C | Breast cancer |  |  | -0.01 | 0.04 | 0.83 | 4 | 20,188 | 0.99 | (0.92 - 1.07) | 0.13 |
| rs11649743 | G | Prostate cancer |  |  | 0.05 | 0.04 | 0.21 | 5 | 22,085 | 1.06 | (0.97 - 1.15) | 0.61 |
| rs11668878 | T | Non-Hodgkin lymphoma (CLL) | | | -0.12 | 0.12 | 0.35 | 1 | 5,275 | 0.89 | (0.7 - 1.14) | . |
| rs11861609 | C | Prostate cancer |  |  | -0.04 | 0.05 | 0.46 | 2 | 10,650 | 0.96 | (0.87 - 1.06) | 0.38 |
| rs12155172 | A | Prostate cancer |  |  | 0.17 | 0.06 | **3.38E-03** | 2 | 10,703 | 1.19 | (1.06 - 1.33) | 0.61 |
| rs1219648 | G | Breast cancer |  |  | 0.02 | 0.04 | 0.65 | 4 | 13,328 | 1.02 | (0.94 - 1.11) | 0.08 |
| rs1229984 | C | Esophageal cancer | |  | 0.00 | 0.12 | 0.97 | 2 | 7,214 | 1 | (0.79 - 1.27) | 0.06 |
| rs12418451 | A | Prostate cancer |  |  | 0.10 | 0.04 | **5.03E-03** | 5 | 22,053 | 1.11 | (1.03 - 1.19) | 0.30 |
| rs12500426 | A | Prostate cancer |  |  | -0.06 | 0.03 | 0.10 | 5 | 22,077 | 0.95 | (0.88 - 1.01) | 0.40 |
| rs12543663 | C | Prostate cancer |  |  | 0.08 | 0.05 | 0.15 | 2 | 10,609 | 1.08 | (0.97 - 1.2) | 0.55 |
| rs12621278 | A | Prostate cancer |  |  | 0.10 | 0.07 | 0.16 | 5 | 22,098 | 1.11 | (0.96 - 1.28) | 0.95 |
| rs13252298 | A | Prostate cancer |  |  | 0.05 | 0.06 | 0.47 | 3 | 8,054 | 1.05 | (0.92 - 1.18) | 0.77 |
| rs13254738 | C | Prostate cancer |  |  | 0.04 | 0.04 | 0.32 | 3 | 15,917 | 1.04 | (0.96 - 1.13) | 0.04 |
| rs13281615 | T | Breast cancer |  |  | 0.10 | 0.03 | **3.44E-03** | 5 | 22,138 | 1.11 | (1.03 - 1.18) | 0.20 |
| rs13387042 | A | Breast cancer |  |  | -0.02 | 0.03 | 0.54 | 5 | 22,109 | 0.98 | (0.92 - 1.05) | 0.76 |
| rs13397985 | G | Non-Hodgkin lymphoma (CLL) | | | -0.07 | 0.06 | 0.22 | 4 | 13,331 | 0.93 | (0.84 - 1.04) | 0.06 |
| rs1412829 | C | Glioma (high-grade) | | | 0.01 | 0.04 | 0.83 | 4 | 13,305 | 1.01 | (0.93 - 1.1) | 0.74 |
| rs1447295 | A | Prostate cancer |  |  | -0.02 | 0.07 | 0.78 | 4 | 13,324 | 0.98 | (0.85 - 1.13) | 0.65 |
| rs1465618 | T | Prostate cancer |  |  | -0.01 | 0.04 | 0.82 | 5 | 22,136 | 0.99 | (0.91 - 1.07) | 0.82 |
| rs1512268 | T | Prostate cancer |  |  | 0.00 | 0.03 | 0.91 | 5 | 22,062 | 1 | (0.93 - 1.06) | 0.01 |
| rs1530057 | A | Lung cancer |  |  | -0.05 | 0.08 | 0.54 | 4 | 20,196 | 0.95 | (0.82 - 1.11) | 0.41 |
| rs1571801 | T | Prostate cancer |  |  | 0.08 | 0.05 | 0.11 | 3 | 14,290 | 1.08 | (0.98 - 1.19) | 0.20 |
| SNP | Coded allele | Previous trait association |  |  | Beta | SE | P-value | # studies | n | OR | 95% CI | Study P-heterogeneity |
| rs157935 | T | Basal cell carcinoma | |  | 0.05 | 0.05 | 0.30 | 4 | 13,331 | 1.05 | (0.96 - 1.15) | 0.92 |
| rs167020 | A | Pancreatic cancer | |  | 0.10 | 0.06 | 0.10 | 2 | 7,217 | 1.11 | (0.98 - 1.24) | 0.76 |
| rs16886165 | G | Breast cancer |  |  | -0.03 | 0.06 | 0.58 | 4 | 13,339 | 0.97 | (0.86 - 1.09) | 0.18 |
| rs16892766 | C | Colorectal cancer | |  | 0.11 | 0.06 | 0.08 | 5 | 22,078 | 1.11 | (0.99 - 1.25) | 0.07 |
| rs16901979 | A | Prostate cancer |  |  | -0.19 | 0.13 | 0.13 | 3 | 14,852 | 0.82 | (0.64 - 1.06) | 0.16 |
| rs16902094 | G | Prostate cancer |  |  | -0.09 | 0.06 | 0.10 | 3 | 16,008 | 0.91 | (0.82 - 1.02) | 0.38 |
| rs17021918 | C | Prostate cancer |  |  | -0.03 | 0.04 | 0.48 | 5 | 22,134 | 0.98 | (0.91 - 1.04) | 0.74 |
| rs172310 | A | Pancreatic cancer | |  | 0.08 | 0.06 | 0.20 | 2 | 7,136 | 1.08 | (0.96 - 1.21) | 0.89 |
| rs17483466 | G | Non-Hodgkin lymphoma (CLL) | | | -0.01 | 0.05 | 0.85 | 4 | 13,333 | 0.99 | (0.89 - 1.1) | 0.25 |
| rs1859962 | G | Prostate cancer |  |  | 0.04 | 0.04 | 0.31 | 4 | 13,338 | 1.04 | (0.96 - 1.13) | 0.38 |
| rs1876206 | G | Breast cancer |  |  | -0.09 | 0.05 | 0.08 | 4 | 20,127 | 0.91 | (0.82 - 1.01) | 0.54 |
| rs189897 | A | Nasopharyngeal carcinoma | | | 0.04 | 0.07 | 0.59 | 2 | 7,217 | 1.04 | (0.9 - 1.2) | 0.81 |
| rs1926203 | T | Lung cancer |  |  | 0.02 | 0.04 | 0.57 | 2 | 13,891 | 1.03 | (0.94 - 1.12) | 0.70 |
| rs1926657 | T | Breast cancer |  |  | -0.04 | 0.05 | 0.38 | 4 | 20,077 | 0.96 | (0.87 - 1.05) | 0.60 |
| rs1978503 | G | Breast cancer |  |  | -0.05 | 0.05 | 0.24 | 4 | 20,185 | 0.95 | (0.87 - 1.04) | 0.33 |
| rs2046210 | A | Breast cancer |  |  | 0.02 | 0.04 | 0.49 | 5 | 22,033 | 1.02 | (0.96 - 1.1) | 0.53 |
| rs2075555 | T | Breast cancer |  |  | -0.10 | 0.08 | 0.24 | 1 | 8,734 | 0.91 | (0.77 - 1.07) | . |
| rs2089222 | A | Leukemia (ALL) |  |  | -0.12 | 0.16 | 0.46 | 2 | 7,220 | 0.89 | (0.65 - 1.21) | 0.05 |
| rs210138 | G | Testicular germ cell tumor | | | -0.07 | 0.05 | 0.23 | 4 | 13,308 | 0.94 | (0.84 - 1.04) | 0.48 |
| rs2151280 | C | Basal cell carcinoma | |  | -0.01 | 0.04 | 0.79 | 4 | 13,301 | 0.99 | (0.91 - 1.08) | 0.29 |
| rs2167364 | C | Leukemia (ALL) |  |  | -0.04 | 0.05 | 0.40 | 4 | 13,334 | 0.96 | (0.88 - 1.05) | 0.41 |
| rs2180341 | G | Breast cancer |  |  | 0.06 | 0.05 | 0.26 | 3 | 11,396 | 1.06 | (0.96 - 1.18) | 0.38 |
| rs2191566 | G | Leukemia (ALL) |  |  | 0.00 | 0.06 | 0.95 | 2 | 7,187 | 1 | (0.88 - 1.12) | 0.93 |
| rs2239633 | G | Leukemia (ALL) |  |  | -0.02 | 0.05 | 0.73 | 2 | 7,207 | 0.98 | (0.88 - 1.09) | 0.45 |
| rs2242041 | G | Leukemia (ALL) |  |  | -0.08 | 0.08 | 0.32 | 4 | 13,332 | 0.93 | (0.8 - 1.08) | 0.78 |
| rs2294008 | T | Bladder cancer |  |  | -0.07 | 0.06 | 0.23 | 2 | 7,203 | 0.94 | (0.84 - 1.04) | 0.90 |
| rs2456449 | G | Non-Hodgkin lymphoma (CLL) | | | 0.00 | 0.05 | 0.92 | 3 | 11,390 | 1 | (0.91 - 1.1) | 0.52 |
| SNP | Coded allele | Previous trait association |  |  | Beta | SE | P-value | # studies | n | OR | 95% CI | Study P-heterogeneity |
| rs2660753 | T | Prostate cancer |  |  | 0.05 | 0.05 | 0.34 | 5 | 22,119 | 1.05 | (0.95 - 1.16) | 0.04 |
| rs266849 | A | Prostate cancer |  |  | 0.02 | 0.05 | 0.64 | 3 | 15,895 | 1.02 | (0.93 - 1.13) | 0.57 |
| rs2710647 | C | Prostate cancer |  |  | -0.04 | 0.03 | 0.20 | 5 | 22,018 | 0.96 | (0.9 - 1.02) | 0.26 |
| rs2735839 | G | Prostate cancer |  |  | 0.01 | 0.05 | 0.82 | 3 | 16,007 | 1.01 | (0.91 - 1.13) | 0.89 |
| rs2736100 | G | Glioma |  |  | -0.01 | 0.03 | 0.78 | 5 | 21,811 | 0.99 | (0.93 - 1.06) | 0.81 |
| rs2808630 | C | Lung cancer |  |  | 0.06 | 0.05 | 0.20 | 4 | 13,335 | 1.06 | (0.97 - 1.17) | 0.85 |
| rs2853676 | A | Glioma |  |  | 0.05 | 0.06 | 0.39 | 2 | 7,172 | 1.05 | (0.94 - 1.19) | 0.85 |
| rs2928679 | A | Prostate cancer |  |  | 0.02 | 0.03 | 0.58 | 5 | 22,083 | 1.02 | (0.95 - 1.09) | 0.08 |
| rs2981578 | G | Breast cancer |  |  | -0.01 | 0.04 | 0.84 | 3 | 15,982 | 0.99 | (0.92 - 1.07) | 0.05 |
| rs2981579 | T | Breast cancer |  |  | 0.04 | 0.05 | 0.45 | 3 | 9,647 | 1.04 | (0.94 - 1.15) | 0.31 |
| rs2981582 | T | Breast cancer |  |  | -0.02 | 0.03 | 0.65 | 5 | 22,114 | 0.98 | (0.92 - 1.05) | 0.27 |
| rs305061 | T | Non-Hodgkin lymphoma (CLL) | | | 0.01 | 0.05 | 0.77 | 3 | 11,396 | 1.01 | (0.92 - 1.12) | 0.52 |
| rs3117582 | C | Lung cancer |  |  | 0.13 | 0.07 | 0.07 | 3 | 11,399 | 1.14 | (0.99 - 1.31) | 0.38 |
| rs3131379 | T | Lung cancer |  |  | 0.02 | 0.05 | 0.75 | 5 | 22,050 | 1.02 | (0.91 - 1.13) | 0.10 |
| rs31489 | C | Lung cancer |  |  | -0.12 | 0.04 | **4.18E-03** | 2 | 14,004 | 0.89 | (0.82 - 0.96) | 0.33 |
| rs3750817 | C | Breast cancer |  |  | 0.00 | 0.04 | 0.96 | 2 | 14,077 | 1 | (0.92 - 1.09) | 0.66 |
| rs3790844 | T | Pancreatic cancer | |  | -0.03 | 0.05 | 0.59 | 3 | 11,400 | 0.97 | (0.87 - 1.08) | 0.01 |
| rs3802842 | C | Colorectal cancer | |  | 0.04 | 0.04 | 0.22 | 5 | 22,122 | 1.05 | (0.97 - 1.12) | 0.67 |
| rs3803662 | T | Breast cancer |  |  | -0.01 | 0.04 | 0.70 | 5 | 22,141 | 0.99 | (0.91 - 1.06) | 0.94 |
| rs3814113 | T | Ovarian cancer |  |  | 0.01 | 0.04 | 0.87 | 3 | 15,981 | 1.01 | (0.93 - 1.09) | 0.22 |
| rs3817198 | C | Breast cancer |  |  | 0.07 | 0.04 | 0.07 | 5 | 22,109 | 1.07 | (1 - 1.15) | 0.89 |
| rs401681 | C | Lung cancer |  |  | -0.14 | 0.03 | **3.65E-05** | 5 | 22,109 | 0.87 | (0.81 - 0.93) | 0.70 |
| rs402710 | C | Lung cancer |  |  | -0.14 | 0.04 | **7.74E-04** | 3 | 15,991 | 0.87 | (0.81 - 0.94) | 0.04 |
| rs4132601 | C | Leukemia (ALL) |  |  | -0.01 | 0.05 | 0.84 | 4 | 13,337 | 0.99 | (0.9 - 1.09) | 0.40 |
| rs4242382 | A | Prostate cancer |  |  | -0.02 | 0.07 | 0.76 | 4 | 13,298 | 0.98 | (0.85 - 1.13) | 0.75 |
| rs4254535 | C | Lung cancer |  |  | -0.01 | 0.05 | 0.78 | 2 | 14,076 | 0.99 | (0.9 - 1.08) | 0.11 |
| rs4295627 | G | Glioma |  |  | -0.02 | 0.07 | 0.75 | 2 | 7,218 | 0.98 | (0.84 - 1.13) | 0.72 |
| rs4324715 | T | Testicular cancer | |  | -0.03 | 0.04 | 0.54 | 4 | 13,290 | 0.97 | (0.9 - 1.06) | 0.09 |
| SNP | Coded allele | Previous trait association |  |  | Beta | SE | P-value | # studies | n | OR | 95% CI | Study P-heterogeneity |
| rs4324798 | A | Lung cancer |  |  | 0.04 | 0.06 | 0.54 | 5 | 21,797 | 1.04 | (0.92 - 1.16) | 0.05 |
| rs4415084 | T | Breast cancer |  |  | 0.00 | 0.04 | 1.00 | 2 | 13,928 | 1 | (0.92 - 1.09) | 0.74 |
| rs4430796 | G | Prostate cancer |  |  | 0.04 | 0.04 | 0.30 | 3 | 15,981 | 1.04 | (0.96 - 1.12) | 0.22 |
| rs4444235 | C | Colorectal cancer | |  | 0.03 | 0.04 | 0.41 | 3 | 16,010 | 1.03 | (0.96 - 1.11) | 0.59 |
| rs445114 | T | Prostate cancer |  |  | 0.04 | 0.06 | 0.48 | 2 | 7,197 | 1.04 | (0.93 - 1.17) | 0.14 |
| rs4474514 | A | Testicular cancer | |  | 0.05 | 0.05 | 0.34 | 4 | 13,327 | 1.05 | (0.95 - 1.17) | 0.66 |
| rs458685 | C | Breast cancer |  |  | 0.05 | 0.06 | 0.43 | 3 | 14,920 | 1.05 | (0.94 - 1.17) | 0.72 |
| rs4624820 | A | Testicular germ cell tumor | | | -0.07 | 0.04 | 0.11 | 4 | 13,324 | 0.94 | (0.86 - 1.02) | 0.14 |
| rs4657482 | A | Testicular germ cell tumor | | | 0.00 | 0.06 | 0.94 | 2 | 7,208 | 1 | (0.89 - 1.12) | 0.86 |
| rs4699052 | C | Testicular germ cell tumor | | | -0.03 | 0.06 | 0.55 | 2 | 7,200 | 0.97 | (0.86 - 1.08) | 0.89 |
| rs4779584 | T | Colorectal cancer | |  | -0.05 | 0.04 | 0.23 | 5 | 22,130 | 0.95 | (0.87 - 1.03) | 0.44 |
| rs4782780 | T | Prostate cancer |  |  | -0.01 | 0.04 | 0.84 | 3 | 14,923 | 0.99 | (0.91 - 1.08) | 0.31 |
| rs4809324 | C | Glioma (high-grade) | | | 0.05 | 0.10 | 0.62 | 1 | 5,276 | 1.05 | (0.86 - 1.28) | . |
| rs4857841 | A | Prostate cancer |  |  | -0.03 | 0.04 | 0.45 | 5 | 22,093 | 0.97 | (0.9 - 1.05) | 0.23 |
| rs4939827 | T | Colorectal cancer | |  | -0.04 | 0.03 | 0.29 | 5 | 22,105 | 0.96 | (0.9 - 1.03) | 0.39 |
| rs4961199 | A | Prostate cancer |  |  | -0.01 | 0.05 | 0.90 | 5 | 22,124 | 0.99 | (0.91 - 1.09) | 0.54 |
| rs4962416 | C | Prostate cancer |  |  | 0.00 | 0.04 | 0.99 | 5 | 22,125 | 1 | (0.93 - 1.08) | 0.22 |
| rs4973768 | T | Breast cancer |  |  | -0.05 | 0.03 | 0.17 | 5 | 22,119 | 0.95 | (0.89 - 1.02) | 0.16 |
| rs4975616 | A | Lung cancer |  |  | -0.14 | 0.03 | **2.30E-05** | 5 | 22,135 | 0.87 | (0.81 - 0.93) | 0.78 |
| rs4977756 | G | Glioma |  |  | -0.03 | 0.04 | 0.46 | 4 | 13,343 | 0.97 | (0.89 - 1.05) | 0.75 |
| rs498872 | T | Glioma |  |  | 0.06 | 0.05 | 0.29 | 3 | 9,629 | 1.06 | (0.95 - 1.17) | 0.45 |
| rs505922 | C | Pancreatic cancer | |  | -0.11 | 0.05 | **0.01** | 4 | 13,339 | 0.89 | (0.82 - 0.98) | 0.40 |
| rs5759167 | G | Prostate cancer |  |  | -0.01 | 0.05 | 0.80 | 2 | 10,681 | 0.99 | (0.9 - 1.09) | 0.00 |
| rs5945572 | A | Prostate cancer |  |  | 0.00 | 0.03 | 0.96 | 3 | 16,014 | 1 | (0.93 - 1.07) | 0.53 |
| rs5945619 | C | Prostate cancer |  |  | 0.00 | 0.03 | 0.90 | 3 | 15,941 | 1 | (0.94 - 1.08) | 0.55 |
| rs6001749 | G | Prostate cancer |  |  | 0.04 | 0.07 | 0.58 | 3 | 8,030 | 1.04 | (0.91 - 1.19) | 0.25 |
| SNP | Coded allele | Previous trait association |  |  | Beta | SE | P-value | # studies | n | OR | 95% CI | Study P-heterogeneity |
| rs6010620 | G | Glioma |  |  | 0.01 | 0.06 | 0.91 | 2 | 7,187 | 1.01 | (0.89 - 1.14) | 0.57 |
| rs620861 | G | Prostate cancer |  |  | 0.09 | 0.05 | 0.07 | 2 | 10,739 | 1.1 | (0.99 - 1.21) | 0.36 |
| rs630014 | C | Pancreatic cancer | |  | -0.01 | 0.04 | 0.76 | 4 | 13,324 | 0.99 | (0.91 - 1.07) | 0.65 |
| rs6435862 | G | Neuroblastoma (high-risk) | | | -0.02 | 0.05 | 0.74 | 4 | 13,254 | 0.98 | (0.9 - 1.08) | 0.33 |
| rs6457327 | C | NHL (Follicular lymphoma) | | | -0.04 | 0.04 | 0.41 | 4 | 13,337 | 0.96 | (0.88 - 1.05) | 0.35 |
| rs6465657 | C | Prostate cancer |  |  | 0.00 | 0.03 | 0.95 | 5 | 22,127 | 1 | (0.94 - 1.07) | 0.45 |
| rs6504950 | G | Breast cancer |  |  | -0.04 | 0.04 | 0.30 | 5 | 22,104 | 0.96 | (0.89 - 1.03) | 0.20 |
| rs6556756 | G | Breast cancer |  |  | -0.09 | 0.06 | 0.10 | 4 | 20,197 | 0.91 | (0.82 - 1.02) | 0.52 |
| rs671 | A | Esophageal cancer | |  | -12.59 | 600.10 | 0.98 | 1 | 7,221 | 0 | . | . |
| rs6939340 | G | Neuroblastoma (high-risk) | | | 0.03 | 0.06 | 0.65 | 2 | 7,177 | 1.03 | (0.92 - 1.14) | 0.24 |
| rs6983267 | G | Colorectal cancer | |  | 0.06 | 0.03 | 0.08 | 5 | 22,097 | 1.06 | (0.99 - 1.13) | 0.71 |
| rs6983561 | C | Prostate cancer |  |  | -0.16 | 0.16 | 0.31 | 2 | 7,216 | 0.85 | (0.63 - 1.16) | 0.52 |
| rs7000448 | T | Prostate cancer |  |  | 0.05 | 0.04 | 0.21 | 4 | 16,833 | 1.05 | (0.97 - 1.14) | 0.13 |
| rs7014346 | A | Colorectal cancer | |  | 0.03 | 0.03 | 0.44 | 5 | 22,097 | 1.03 | (0.96 - 1.1) | 0.68 |
| rs7089424 | C | Leukemia (ALL) |  |  | 0.05 | 0.06 | 0.39 | 2 | 7,212 | 1.05 | (0.94 - 1.18) | 0.71 |
| rs710521 | A | Urinary bladder cancer | | | 0.13 | 0.06 | **0.03** | 3 | 9,608 | 1.13 | (1.01 - 1.27) | 0.55 |
| rs7117034 | T | Prostate cancer |  |  | 0.21 | 0.06 | **3.67E-04** | 2 | 10,675 | 1.23 | (1.1 - 1.37) | 0.55 |
| rs7127900 | A | Prostate cancer |  |  | -0.03 | 0.07 | 0.67 | 2 | 7,193 | 0.97 | (0.85 - 1.11) | 0.76 |
| rs7176508 | A | Non-Hodgkin lymphoma (CLL) | | | 0.10 | 0.05 | **0.04** | 3 | 11,395 | 1.1 | (1 - 1.21) | 0.78 |
| rs719725 | A | Colorectal cancer | |  | 0.04 | 0.03 | 0.23 | 5 | 22,041 | 1.04 | (0.97 - 1.12) | 0.48 |
| rs721048 | A | Prostate cancer |  |  | 0.08 | 0.07 | 0.28 | 2 | 7,201 | 1.08 | (0.94 - 1.24) | 0.08 |
| rs735665 | A | Non-Hodgkin lymphoma (CLL) | | | 0.00 | 0.05 | 0.98 | 4 | 13,323 | 1 | (0.9 - 1.11) | 0.17 |
| rs748404 | T | Lung cancer |  |  | 0.02 | 0.04 | 0.57 | 4 | 20,099 | 1.02 | (0.94 - 1.11) | 0.48 |
| rs7501939 | C | Prostate cancer |  |  | 0.00 | 0.04 | 0.92 | 3 | 15,985 | 1 | (0.93 - 1.09) | 0.96 |
| rs7538876 | A | Basal cell carcinoma | |  | 0.04 | 0.04 | 0.39 | 4 | 13,327 | 1.04 | (0.95 - 1.13) | 0.93 |
| rs757978 | A | Non-Hodgkin lymphoma (CLL) | | | -0.06 | 0.08 | 0.41 | 3 | 11,399 | 0.94 | (0.81 - 1.09) | 0.02 |
| rs7626795 | G | Lung cancer |  |  | -0.07 | 0.05 | 0.17 | 4 | 19,643 | 0.93 | (0.83 - 1.03) | 0.11 |
| SNP | Coded allele | Previous trait association |  |  | Beta | SE | P-value | # studies | n | OR | 95% CI | Study P-heterogeneity |
| rs7679673 | C | Prostate cancer |  |  | -0.04 | 0.04 | 0.32 | 3 | 15,908 | 0.96 | (0.89 - 1.04) | 0.42 |
| rs7809758 | G | Leukemia (ALL) |  |  | -0.02 | 0.04 | 0.62 | 4 | 13,337 | 0.98 | (0.9 - 1.07) | 0.33 |
| rs7837688 | T | Prostate cancer |  |  | -0.07 | 0.07 | 0.32 | 3 | 16,018 | 0.94 | (0.82 - 1.07) | 0.55 |
| rs7841060 | G | Prostate cancer |  |  | 0.01 | 0.05 | 0.78 | 3 | 15,970 | 1.01 | (0.92 - 1.11) | 0.03 |
| rs7931342 | G | Prostate cancer |  |  | 0.03 | 0.04 | 0.48 | 4 | 13,336 | 1.03 | (0.95 - 1.12) | 0.68 |
| rs801114 | G | Basal cell carcinoma | |  | 0.00 | 0.04 | 0.91 | 4 | 13,340 | 1 | (0.91 - 1.09) | 0.90 |
| rs8034191 | C | Lung cancer |  |  | -0.03 | 0.04 | 0.53 | 4 | 13,335 | 0.97 | (0.89 - 1.06) | 0.06 |
| rs8042374 | G | Lung cancer |  |  | -0.04 | 0.04 | 0.35 | 5 | 22,032 | 0.96 | (0.89 - 1.04) | 0.01 |
| rs8102476 | C | Prostate cancer |  |  | 0.01 | 0.05 | 0.83 | 2 | 10,743 | 1.01 | (0.92 - 1.11) | 0.18 |
| rs872071 | G | Non-Hodgkin lymphoma (CLL) | | | 0.08 | 0.06 | 0.17 | 2 | 7,210 | 1.08 | (0.97 - 1.2) | 0.95 |
| rs889312 | C | Breast cancer |  |  | 0.04 | 0.04 | 0.25 | 5 | 22,098 | 1.04 | (0.97 - 1.12) | 0.31 |
| rs9295740 | A | Lung cancer |  |  | -0.08 | 0.04 | 0.06 | 5 | 22,134 | 0.92 | (0.84 - 1) | 0.34 |
| rs931794 | G | Lung cancer |  |  | -0.04 | 0.04 | 0.26 | 5 | 22,041 | 0.96 | (0.9 - 1.03) | 0.07 |
| rs9364554 | T | Prostate cancer |  |  | 0.02 | 0.04 | 0.60 | 5 | 22,117 | 1.02 | (0.95 - 1.1) | 0.01 |
| rs944289 | T | Thyroid cancer |  |  | 0.01 | 0.05 | 0.82 | 3 | 10,831 | 1.01 | (0.92 - 1.11) | 0.24 |
| rs9543325 | C | Pancreatic cancer | |  | -0.09 | 0.05 | 0.07 | 3 | 11,397 | 0.92 | (0.83 - 1.01) | 0.96 |
| rs961253 | A | Colorectal cancer | |  | 0.03 | 0.04 | 0.46 | 5 | 22,119 | 1.03 | (0.96 - 1.1) | 0.80 |
| rs9623117 | C | Prostate cancer |  |  | 0.01 | 0.04 | 0.81 | 4 | 20,166 | 1.01 | (0.93 - 1.1) | 0.78 |
| rs9642880 | T | Urinary bladder cancer | | | -0.03 | 0.05 | 0.48 | 3 | 11,396 | 0.97 | (0.88 - 1.06) | 0.87 |
| rs965513 | A | Thyroid cancer |  |  | -0.01 | 0.05 | 0.78 | 4 | 13,330 | 0.99 | (0.9 - 1.08) | 0.64 |
| rs981782 | T | Breast cancer |  |  | -0.04 | 0.04 | 0.36 | 3 | 16,004 | 0.97 | (0.89 - 1.04) | 0.43 |
| rs9929218 | G | Colorectal cancer | |  | -0.05 | 0.04 | 0.19 | 5 | 22,140 | 0.95 | (0.89 - 1.02) | 0.87 |
| rs995030 | G | Testicular germ cell tumor | | | 0.06 | 0.05 | 0.25 | 4 | 13,327 | 1.07 | (0.96 - 1.19) | 0.56 |
| rs999737 | C | Breast cancer |  |  | -0.02 | 0.05 | 0.61 | 2 | 13,976 | 0.98 | (0.89 - 1.07) | 0.33 |
