# Supplementary material for: Pleiotropic and Sex-Specific Effects of Cancer GWAS SNPs on Melanoma Risk in the Population Architecture Using Genomics and Epidemiology (PAGE) Study
Source: PLoS One. 2015 Mar 19;10(3):e0120491. doi: 10.1371/journal.pone.0120491 (PMC4366224; doi:10.1371/journal.pone.0120491)
Supplement: S3 Table — Results for the association between melanoma and each of the 181 SNPs, stratified by sex. (DOCX) [file pone.0120491.s004.docx]

Kocarnik et al.

Evaluation of pleiotropic and sex-specific effects of cancer GWAS SNPs on melanoma risk in the Population Architectures using Genomics and Epidemiology (PAGE) study

| **Table S3 - Results for the association between melanoma and each of the 181 SNPs, stratified by sex.** SNPs are in numerical order, and P-values for the association with melanoma < 0.05 and P-heterogeneity < 0.05 are in bold. | | | | | | | | | |
| --- | --- | --- | --- | --- | --- | --- | --- | --- | --- |
| SNP | Previous trait association | Sex | OR | 95% CI | P-value | # studies | n | Study P-heterogeneity | Sex P-heterogeneity |
| rs10086908 | Prostate cancer | Male | 0.92 | (0.82 - 1.04) | 0.19 | 3 | 8,234 | 0.29 | 0.28 |
|  |  | Female | 1.01 | (0.9 - 1.15) | 0.81 | 3 | 8,606 | 0.39 |  |
| rs10090154 | Prostate cancer | Male | 0.93 | (0.74 - 1.17) | 0.54 | 2 | 5,707 | 0.21 | 0.69 |
|  |  | Female | 1 | (0.78 - 1.28) | 1.00 | 2 | 4,898 | 0.89 |  |
| rs1016343 | Prostate cancer | Male | 1.37 | (0.99 - 1.91) | 0.06 | 1 | 1,019 | . | 0.20 |
|  |  | Female | 1.08 | (0.94 - 1.25) | 0.28 | 2 | 6,169 | 0.68 |  |
| rs10220831 | Non-Hodgkin lymphoma (CLL) | Male | 1.1 | (0.92 - 1.32) | 0.29 | 2 | 3,403 | 0.83 | 0.40 |
|  |  | Female | 0.99 | (0.85 - 1.16) | 0.95 | 2 | 4,584 | 0.07 |  |
| rs10263639 | Breast cancer | Male | 0.73 | (0.52 - 1.03) | 0.07 | 1 | 2,428 | . | **0.04** |
|  |  | Female | 1.07 | (0.94 - 1.23) | 0.32 | 2 | 8,964 | 0.90 |  |
| rs1036935 | Non-Hodgkin lymphoma (CLL) | Male | 0.72 | (0.54 - 0.96) | **0.03** | 1 | 2,428 | . | **0.02** |
|  |  | Female | 1.06 | (0.94 - 1.19) | 0.32 | 2 | 8,971 | 0.48 |  |
| rs10411210 | Colorectal cancer | Male | 1.03 | (0.84 - 1.28) | 0.75 | 2 | 5,797 | 0.58 | 0.27 |
|  |  | Female | 1.2 | (1.02 - 1.42) | **0.03** | 3 | 10,174 | 0.84 |  |
| rs1045485 | Breast cancer | Male | 0.83 | (0.71 - 0.96) | **0.02** | 3 | 8,242 | 0.22 | 0.11 |
|  |  | Female | 0.99 | (0.85 - 1.15) | 0.88 | 4 | 12,776 | 0.03 |  |
| rs10464870 | Glioma | Male | 1.09 | (0.77 - 1.53) | 0.64 | 1 | 1,016 | . | 0.67 |
|  |  | Female | 1 | (0.87 - 1.16) | 1.00 | 2 | 6,192 | 0.22 |  |
| rs10483813 | Breast cancer | Male | 0.93 | (0.75 - 1.14) | 0.46 | 2 | 3,451 | 0.70 | 0.47 |
|  |  | Female | 1.02 | (0.86 - 1.22) | 0.81 | 2 | 4,606 | 0.03 |  |
| SNP | Previous trait association | Sex | OR | 95% CI | P-value | # studies | n | Study P-heterogeneity | Sex P-heterogeneity |
| rs10486567 | Prostate cancer | Male | 1.14 | (1 - 1.31) | **0.05** | 3 | 8,228 | 0.48 | 0.24 |
|  |  | Female | 1.03 | (0.92 - 1.16) | 0.62 | 3 | 10,199 | 0.04 |  |
| rs10490113 | Breast cancer | Male | 1.03 | (0.86 - 1.24) | 0.74 | 2 | 7,175 | 0.61 | 0.86 |
|  |  | Female | 1.01 | (0.88 - 1.16) | 0.87 | 3 | 12,947 | 0.92 |  |
| rs10505477 | Colorectal cancer | Male | 1.06 | (0.95 - 1.18) | 0.33 | 3 | 8,232 | 0.99 | 0.37 |
|  |  | Female | 0.97 | (0.83 - 1.13) | 0.68 | 2 | 4,912 | 0.55 |  |
| rs1051730 | Lung cancer | Male | 0.95 | (0.85 - 1.07) | 0.41 | 3 | 8,244 | 0.16 | 0.42 |
|  |  | Female | 1.01 | (0.93 - 1.1) | 0.81 | 4 | 13,883 | 0.90 |  |
| rs10778826 | Prostate cancer | Male | 1.07 | (0.96 - 1.19) | 0.25 | 3 | 8,245 | 0.17 | 0.43 |
|  |  | Female | 1.01 | (0.93 - 1.1) | 0.84 | 4 | 13,889 | 0.73 |  |
| rs10795668 | Colorectal cancer | Male | 1.04 | (0.92 - 1.17) | 0.53 | 3 | 8,171 | 0.08 | 0.72 |
|  |  | Female | 1.01 | (0.93 - 1.1) | 0.81 | 4 | 13,850 | 0.94 |  |
| rs10821936 | Leukemia (ALL) | Male | 0.99 | (0.83 - 1.19) | 0.95 | 2 | 3,453 | 0.68 | 0.62 |
|  |  | Female | 1.05 | (0.95 - 1.16) | 0.36 | 3 | 9,880 | 0.91 |  |
| rs10896449 | Prostate cancer | Male | 1.1 | (0.99 - 1.23) | 0.07 | 3 | 8,221 | 0.86 | 0.28 |
|  |  | Female | 1.02 | (0.94 - 1.11) | 0.58 | 4 | 13,871 | 0.34 |  |
| rs10941679 | Breast cancer | Male | 0.86 | (0.63 - 1.19) | 0.38 | 1 | 1,026 | . | 0.63 |
|  |  | Female | 0.94 | (0.82 - 1.08) | 0.39 | 2 | 6,181 | 0.91 |  |
| rs10974944 | Leukemia (Myeloid) | Male | 0.98 | (0.81 - 1.18) | 0.84 | 2 | 3,455 | 0.38 | 0.82 |
|  |  | Female | 0.96 | (0.86 - 1.07) | 0.43 | 3 | 9,886 | 0.21 |  |
| rs10993994 | Prostate cancer | Male | 0.89 | (0.78 - 1.01) | 0.07 | 2 | 5,817 | 0.00 | **0.03** |
|  |  | Female | 1.06 | (0.96 - 1.17) | 0.24 | 3 | 10,198 | 0.97 |  |
| rs10994982 | Leukemia (ALL) | Male | 0.98 | (0.83 - 1.17) | 0.86 | 2 | 3,434 | 0.52 | 0.38 |
|  |  | Female | 1.08 | (0.98 - 1.18) | 0.13 | 3 | 9,873 | 0.74 |  |
| rs11083846 | Non-Hodgkin lymphoma (CLL) | Male | 0.99 | (0.71 - 1.36) | 0.93 | 1 | 1,025 | . | 0.90 |
|  |  | Female | 0.96 | (0.84 - 1.11) | 0.61 | 2 | 6,189 | 0.88 |  |
| rs11155133 | Leukemia (ALL) | Male | 0.58 | (0.14 - 2.41) | 0.45 | 1 | 1,024 | . | 0.72 |
|  |  | Female | 0.77 | (0.41 - 1.44) | 0.42 | 2 | 6,195 | 0.75 |  |
| SNP | Previous trait association | Sex | OR | 95% CI | P-value | # studies | n | Study P-heterogeneity | Sex P-heterogeneity |
| rs11170164 | Basal cell carcinoma | Male | 0.82 | (0.45 - 1.48) | 0.51 | 1 | 1,017 | . | 0.57 |
|  |  | Female | 1.06 | (0.54 - 2.05) | 0.87 | 1 | 908 | . |  |
| rs11228565 | Prostate cancer | Male | 1.21 | (1.03 - 1.42) | **0.02** | 1 | 4,756 | . | 0.05 |
|  |  | Female | 0.99 | (0.87 - 1.12) | 0.84 | 2 | 9,270 | 0.00 |  |
| rs11249433 | Breast cancer | Male | 1 | (0.88 - 1.13) | 0.95 | 2 | 5,818 | 0.74 | 0.98 |
|  |  | Female | 0.99 | (0.9 - 1.1) | 0.90 | 3 | 9,794 | 0.21 |  |
| rs11649338 | Breast cancer | Male | 1.02 | (0.9 - 1.15) | 0.79 | 2 | 7,215 | 0.49 | 0.62 |
|  |  | Female | 0.98 | (0.9 - 1.07) | 0.65 | 3 | 12,973 | 0.03 |  |
| rs11649743 | Prostate cancer | Male | 1.16 | (1.01 - 1.35) | **0.04** | 3 | 8,207 | 0.26 | 0.10 |
|  |  | Female | 1 | (0.9 - 1.11) | 0.98 | 4 | 13,878 | 0.93 |  |
| rs11668878 | Non-Hodgkin lymphoma (CLL) | Male | . | . | . | 0 | 0 | . | . |
|  |  | Female | 0.89 | (0.7 - 1.14) | 0.35 | 1 | 5,275 | . |  |
| rs11861609 | Prostate cancer | Male | 0.96 | (0.84 - 1.09) | 0.48 | 2 | 5,773 | 0.45 | 0.80 |
|  |  | Female | 0.98 | (0.84 - 1.14) | 0.80 | 2 | 4,877 | 0.55 |  |
| rs12155172 | Prostate cancer | Male | 1.26 | (1.09 - 1.46) | **1.66E-03** | 2 | 5,806 | 0.66 | 0.18 |
|  |  | Female | 1.08 | (0.9 - 1.29) | 0.43 | 2 | 4,897 | 0.81 |  |
| rs1219648 | Breast cancer | Male | 1.16 | (0.97 - 1.38) | 0.10 | 2 | 3,444 | 0.03 | 0.10 |
|  |  | Female | 0.98 | (0.89 - 1.08) | 0.69 | 3 | 9,884 | 0.69 |  |
| rs1229984 | Esophageal cancer | Male | 2.5 | (1.11 - 5.63) | 0.03 | 1 | 1,024 | . | **0.02** |
|  |  | Female | 0.9 | (0.7 - 1.15) | 0.40 | 2 | 6,190 | 0.91 |  |
| rs12418451 | Prostate cancer | Male | 1.22 | (1.09 - 1.37) | **7.96E-04** | 3 | 8,213 | 0.21 | **0.04** |
|  |  | Female | 1.05 | (0.96 - 1.14) | 0.33 | 4 | 13,840 | 0.42 |  |
| rs12500426 | Prostate cancer | Male | 0.93 | (0.83 - 1.03) | 0.17 | 3 | 8,243 | 0.26 | 0.63 |
|  |  | Female | 0.96 | (0.88 - 1.04) | 0.32 | 4 | 13,834 | 0.10 |  |
| rs12543663 | Prostate cancer | Male | 1.15 | (1.01 - 1.31) | **0.04** | 2 | 5,707 | 0.69 | 0.15 |
|  |  | Female | 0.98 | (0.83 - 1.16) | 0.83 | 2 | 4,902 | 0.14 |  |
| rs12621278 | Prostate cancer | Male | 1.13 | (0.89 - 1.44) | 0.32 | 3 | 8,233 | 0.75 | 0.83 |
|  |  | Female | 1.1 | (0.91 - 1.31) | 0.33 | 4 | 13,865 | 0.92 |  |
| SNP | Previous trait association | Sex | OR | 95% CI | P-value | # studies | n | Study P-heterogeneity | Sex P-heterogeneity |
| rs13252298 | Prostate cancer | Male | 1.15 | (0.94 - 1.39) | 0.17 | 2 | 3,447 | 0.60 | 0.22 |
|  |  | Female | 0.98 | (0.83 - 1.15) | 0.79 | 2 | 4,607 | 0.60 |  |
| rs13254738 | Prostate cancer | Male | 1.06 | (0.93 - 1.21) | 0.38 | 2 | 5,783 | 0.00 | 0.74 |
|  |  | Female | 1.03 | (0.93 - 1.14) | 0.56 | 3 | 10,134 | 0.18 |  |
| rs13281615 | Breast cancer | Male | 1.12 | (1 - 1.25) | 0.05 | 3 | 8,246 | 0.34 | 0.84 |
|  |  | Female | 1.1 | (1.01 - 1.2) | **0.03** | 4 | 13,892 | 0.27 |  |
| rs13387042 | Breast cancer | Male | 1 | (0.9 - 1.12) | 0.96 | 3 | 8,234 | 0.59 | 0.59 |
|  |  | Female | 0.97 | (0.89 - 1.05) | 0.41 | 4 | 13,875 | 0.69 |  |
| rs13397985 | Non-Hodgkin lymphoma (CLL) | Male | 0.86 | (0.69 - 1.08) | 0.20 | 2 | 3,453 | 0.14 | 0.41 |
|  |  | Female | 0.96 | (0.85 - 1.09) | 0.52 | 3 | 9,878 | 0.03 |  |
| rs1412829 | Glioma (high-grade) | Male | 1.08 | (0.9 - 1.29) | 0.40 | 2 | 3,432 | 0.81 | 0.40 |
|  |  | Female | 0.99 | (0.9 - 1.09) | 0.82 | 3 | 9,873 | 0.67 |  |
| rs1447295 | Prostate cancer | Male | 0.98 | (0.73 - 1.32) | 0.90 | 2 | 3,450 | 0.77 | 0.99 |
|  |  | Female | 0.98 | (0.84 - 1.15) | 0.83 | 3 | 9,874 | 0.39 |  |
| rs1465618 | Prostate cancer | Male | 0.95 | (0.83 - 1.09) | 0.47 | 3 | 8,244 | 0.84 | 0.45 |
|  |  | Female | 1.02 | (0.92 - 1.12) | 0.77 | 4 | 13,892 | 0.50 |  |
| rs1512268 | Prostate cancer | Male | 0.99 | (0.89 - 1.11) | 0.92 | 3 | 8,216 | 0.03 | 0.98 |
|  |  | Female | 1 | (0.92 - 1.08) | 0.94 | 4 | 13,846 | 0.01 |  |
| rs1530057 | Lung cancer | Male | 1.07 | (0.84 - 1.38) | 0.58 | 2 | 7,220 | 0.39 | 0.24 |
|  |  | Female | 0.89 | (0.73 - 1.08) | 0.23 | 3 | 12,976 | 0.61 |  |
| rs1571801 | Prostate cancer | Male | 1.05 | (0.91 - 1.21) | 0.55 | 2 | 5,696 | 0.01 | 0.49 |
|  |  | Female | 1.12 | (0.99 - 1.26) | 0.08 | 3 | 8,594 | 0.06 |  |
| rs157935 | Basal cell carcinoma | Male | 0.99 | (0.82 - 1.2) | 0.94 | 2 | 3,447 | 0.79 | 0.50 |
|  |  | Female | 1.07 | (0.96 - 1.19) | 0.22 | 3 | 9,884 | 0.78 |  |
| rs167020 | Pancreatic cancer | Male | 1.2 | (0.89 - 1.63) | 0.24 | 1 | 1,028 | . | 0.56 |
|  |  | Female | 1.09 | (0.96 - 1.24) | 0.19 | 2 | 6,189 | 0.85 |  |
| SNP | Previous trait association | Sex | OR | 95% CI | P-value | # studies | n | Study P-heterogeneity | Sex P-heterogeneity |
| rs16886165 | Breast cancer | Male | 0.99 | (0.78 - 1.25) | 0.94 | 2 | 3,452 | 0.01 | 0.86 |
|  |  | Female | 0.97 | (0.85 - 1.1) | 0.62 | 3 | 9,887 | 0.77 |  |
| rs16892766 | Colorectal cancer | Male | 1.34 | (1.11 - 1.61) | **2.17E-03** | 3 | 8,221 | 0.16 | **0.01** |
|  |  | Female | 0.98 | (0.84 - 1.15) | 0.82 | 4 | 13,857 | 0.93 |  |
| rs16901979 | Prostate cancer | Male | 0.87 | (0.62 - 1.23) | 0.45 | 2 | 7,194 | 0.09 | 0.63 |
|  |  | Female | 0.77 | (0.54 - 1.11) | 0.16 | 2 | 7,658 | 0.45 |  |
| rs16902094 | Prostate cancer | Male | 0.98 | (0.82 - 1.17) | 0.81 | 2 | 5,813 | 0.40 | 0.33 |
|  |  | Female | 0.87 | (0.76 - 1.01) | 0.06 | 3 | 10,195 | 0.37 |  |
| rs17021918 | Prostate cancer | Male | 0.96 | (0.86 - 1.07) | 0.47 | 3 | 8,244 | 0.57 | 0.75 |
|  |  | Female | 0.98 | (0.9 - 1.07) | 0.68 | 4 | 13,890 | 0.27 |  |
| rs172310 | Pancreatic cancer | Male | 1.09 | (0.81 - 1.47) | 0.57 | 1 | 1,025 | . | 0.94 |
|  |  | Female | 1.08 | (0.95 - 1.22) | 0.25 | 2 | 6,111 | 0.78 |  |
| rs17483466 | Non-Hodgkin lymphoma (CLL) | Male | 0.97 | (0.79 - 1.2) | 0.77 | 2 | 3,448 | 0.72 | 0.82 |
|  |  | Female | 1 | (0.89 - 1.12) | 0.97 | 3 | 9,885 | 0.13 |  |
| rs1859962 | Prostate cancer | Male | 1.05 | (0.88 - 1.24) | 0.59 | 2 | 3,455 | 0.59 | 0.97 |
|  |  | Female | 1.04 | (0.95 - 1.15) | 0.38 | 3 | 9,883 | 0.10 |  |
| rs1876206 | Breast cancer | Male | 0.87 | (0.73 - 1.04) | 0.12 | 2 | 7,196 | 0.92 | 0.52 |
|  |  | Female | 0.93 | (0.82 - 1.06) | 0.28 | 3 | 12,931 | 0.39 |  |
| rs189897 | Nasopharyngeal carcinoma | Male | 1.15 | (0.81 - 1.63) | 0.44 | 1 | 1,025 | . | 0.55 |
|  |  | Female | 1.02 | (0.88 - 1.19) | 0.78 | 2 | 6,192 | 0.31 |  |
| rs1926203 | Lung cancer | Male | 1.05 | (0.9 - 1.22) | 0.54 | 1 | 4,702 | . | 0.70 |
|  |  | Female | 1.01 | (0.91 - 1.12) | 0.82 | 2 | 9,189 | 0.42 |  |
| rs1926657 | Breast cancer | Male | 0.97 | (0.83 - 1.13) | 0.68 | 2 | 7,188 | 0.47 | 0.90 |
|  |  | Female | 0.95 | (0.85 - 1.07) | 0.43 | 3 | 12,889 | 0.39 |  |
| rs1978503 | Breast cancer | Male | 1 | (0.86 - 1.16) | 0.98 | 2 | 7,211 | 0.55 | 0.42 |
|  |  | Female | 0.92 | (0.83 - 1.03) | 0.16 | 3 | 12,974 | 0.28 |  |
| SNP | Previous trait association | Sex | OR | 95% CI | P-value | # studies | n | Study P-heterogeneity | Sex P-heterogeneity |
| rs2046210 | Breast cancer | Male | 0.97 | (0.87 - 1.09) | 0.60 | 3 | 8,158 | 0.86 | 0.24 |
|  |  | Female | 1.06 | (0.97 - 1.15) | 0.21 | 4 | 13,875 | 0.54 |  |
| rs2075555 | Breast cancer | Male | 0.98 | (0.8 - 1.22) | 0.89 | 1 | 4,757 | . | 0.24 |
|  |  | Female | 0.8 | (0.61 - 1.05) | 0.11 | 1 | 3,977 | . |  |
| rs2089222 | Leukemia (ALL) | Male | 0.49 | (0.2 - 1.23) | 0.13 | 1 | 1,025 | . | 0.17 |
|  |  | Female | 0.96 | (0.69 - 1.34) | 0.83 | 2 | 6,195 | 0.18 |  |
| rs210138 | Testicular germ cell tumor | Male | 0.88 | (0.71 - 1.1) | 0.26 | 2 | 3,437 | 0.57 | 0.54 |
|  |  | Female | 0.95 | (0.84 - 1.08) | 0.44 | 3 | 9,871 | 0.33 |  |
| rs2151280 | Basal cell carcinoma | Male | 0.95 | (0.8 - 1.13) | 0.57 | 2 | 3,433 | 0.37 | 0.62 |
|  |  | Female | 1 | (0.91 - 1.1) | 1.00 | 3 | 9,868 | 0.12 |  |
| rs2167364 | Leukemia (ALL) | Male | 1.01 | (0.84 - 1.21) | 0.92 | 2 | 3,452 | 0.69 | 0.57 |
|  |  | Female | 0.95 | (0.86 - 1.05) | 0.32 | 3 | 9,882 | 0.27 |  |
| rs2180341 | Breast cancer | Male | 0.93 | (0.72 - 1.2) | 0.58 | 1 | 2,428 | . | 0.27 |
|  |  | Female | 1.09 | (0.97 - 1.22) | 0.14 | 2 | 8,968 | 0.40 |  |
| rs2191566 | Leukemia (ALL) | Male | 0.87 | (0.64 - 1.18) | 0.37 | 1 | 1,026 | . | 0.34 |
|  |  | Female | 1.02 | (0.9 - 1.16) | 0.76 | 2 | 6,161 | 0.28 |  |
| rs2239633 | Leukemia (ALL) | Male | 0.76 | (0.58 - 1.01) | 0.06 | 1 | 1,025 | . | 0.06 |
|  |  | Female | 1.02 | (0.91 - 1.15) | 0.70 | 2 | 6,182 | 0.38 |  |
| rs2242041 | Leukemia (ALL) | Male | 0.98 | (0.72 - 1.34) | 0.90 | 2 | 3,448 | 0.40 | 0.69 |
|  |  | Female | 0.91 | (0.77 - 1.08) | 0.30 | 3 | 9,884 | 0.63 |  |
| rs2294008 | Bladder cancer | Male | 0.75 | (0.56 - 1) | 0.05 | 1 | 1,015 | . | 0.11 |
|  |  | Female | 0.97 | (0.86 - 1.09) | 0.62 | 2 | 6,188 | 0.13 |  |
| rs2456449 | Non-Hodgkin lymphoma (CLL) | Male | 0.89 | (0.7 - 1.12) | 0.32 | 1 | 2,428 | . | 0.26 |
|  |  | Female | 1.03 | (0.93 - 1.14) | 0.59 | 2 | 8,962 | 0.96 |  |
| rs2660753 | Prostate cancer | Male | 1.24 | (1.06 - 1.46) | **0.01** | 3 | 8,232 | 0.36 | **0.01** |
|  |  | Female | 0.94 | (0.82 - 1.08) | 0.38 | 4 | 13,887 | 0.39 |  |
| SNP | Previous trait association | Sex | OR | 95% CI | P-value | # studies | n | Study P-heterogeneity | Sex P-heterogeneity |
| rs266849 | Prostate cancer | Male | 0.95 | (0.82 - 1.12) | 0.56 | 2 | 5,731 | 0.95 | 0.28 |
|  |  | Female | 1.07 | (0.94 - 1.21) | 0.31 | 3 | 10,164 | 0.13 |  |
| rs2710647 | Prostate cancer | Male | 0.97 | (0.87 - 1.08) | 0.55 | 3 | 8,177 | 0.21 | 0.82 |
|  |  | Female | 0.95 | (0.88 - 1.03) | 0.24 | 4 | 13,841 | 0.52 |  |
| rs2735839 | Prostate cancer | Male | 1 | (0.84 - 1.19) | 0.97 | 2 | 5,807 | 0.51 | 0.90 |
|  |  | Female | 1.02 | (0.89 - 1.16) | 0.80 | 3 | 10,200 | 0.90 |  |
| rs2736100 | Glioma | Male | 0.94 | (0.84 - 1.05) | 0.26 | 3 | 8,120 | 0.60 | 0.23 |
|  |  | Female | 1.02 | (0.94 - 1.11) | 0.60 | 4 | 13,691 | 0.73 |  |
| rs2808630 | Lung cancer | Male | 1 | (0.83 - 1.21) | 1.00 | 2 | 3,446 | 0.84 | 0.48 |
|  |  | Female | 1.08 | (0.97 - 1.2) | 0.14 | 3 | 9,889 | 0.85 |  |
| rs2853676 | Glioma | Male | 1.02 | (0.75 - 1.4) | 0.89 | 1 | 999 | . | 0.84 |
|  |  | Female | 1.06 | (0.93 - 1.21) | 0.38 | 2 | 6,173 | 0.94 |  |
| rs2928679 | Prostate cancer | Male | 1.14 | (1.02 - 1.27) | **0.02** | 3 | 8,218 | 0.21 | **0.01** |
|  |  | Female | 0.96 | (0.88 - 1.04) | 0.30 | 4 | 13,865 | 0.91 |  |
| rs2981578 | Breast cancer | Male | 1 | (0.88 - 1.13) | 0.95 | 2 | 5,808 | 0.02 | 0.94 |
|  |  | Female | 0.99 | (0.9 - 1.09) | 0.84 | 3 | 10,174 | 0.52 |  |
| rs2981579 | Breast cancer | Male | 1.12 | (0.94 - 1.33) | 0.21 | 2 | 3,455 | 0.12 | 0.32 |
|  |  | Female | 1 | (0.89 - 1.13) | 0.94 | 2 | 6,192 | 0.80 |  |
| rs2981582 | Breast cancer | Male | 1.02 | (0.91 - 1.14) | 0.70 | 3 | 8,232 | 0.09 | 0.40 |
|  |  | Female | 0.96 | (0.88 - 1.05) | 0.39 | 4 | 13,882 | 0.85 |  |
| rs305061 | Non-Hodgkin lymphoma (CLL) | Male | 0.97 | (0.77 - 1.21) | 0.77 | 1 | 2,428 | . | 0.64 |
|  |  | Female | 1.03 | (0.92 - 1.14) | 0.65 | 2 | 8,968 | 0.29 |  |
| rs3117582 | Lung cancer | Male | 1.39 | (1.02 - 1.91) | **0.04** | 1 | 2,428 | . | 0.16 |
|  |  | Female | 1.08 | (0.93 - 1.27) | 0.32 | 2 | 8,971 | 0.95 |  |
| rs3131379 | Lung cancer | Male | 0.95 | (0.79 - 1.14) | 0.56 | 3 | 8,197 | 0.01 | 0.31 |
|  |  | Female | 1.07 | (0.93 - 1.22) | 0.35 | 4 | 13,853 | 0.92 |  |
| SNP | Previous trait association | Sex | OR | 95% CI | P-value | # studies | n | Study P-heterogeneity | Sex P-heterogeneity |
| rs31489 | Lung cancer | Male | 0.91 | (0.79 - 1.05) | 0.19 | 1 | 4,764 | . | 0.67 |
|  |  | Female | 0.88 | (0.79 - 0.97) | **0.01** | 2 | 9,240 | 0.11 |  |
| rs3750817 | Breast cancer | Male | 0.98 | (0.84 - 1.13) | 0.73 | 1 | 4,794 | . | 0.65 |
|  |  | Female | 1.02 | (0.92 - 1.13) | 0.77 | 2 | 9,283 | 0.83 |  |
| rs3790844 | Pancreatic cancer | Male | 0.95 | (0.74 - 1.22) | 0.68 | 1 | 2,428 | . | 0.84 |
|  |  | Female | 0.98 | (0.87 - 1.1) | 0.68 | 2 | 8,972 | 2.77E-03 |  |
| rs3802842 | Colorectal cancer | Male | 0.99 | (0.87 - 1.11) | 0.81 | 3 | 8,241 | 0.95 | 0.22 |
|  |  | Female | 1.08 | (0.99 - 1.18) | 0.09 | 4 | 13,881 | 0.81 |  |
| rs3803662 | Breast cancer | Male | 1.04 | (0.92 - 1.17) | 0.52 | 3 | 8,243 | 0.97 | 0.26 |
|  |  | Female | 0.95 | (0.87 - 1.05) | 0.31 | 4 | 13,898 | 0.92 |  |
| rs3814113 | Ovarian cancer | Male | 0.93 | (0.81 - 1.06) | 0.26 | 2 | 5,807 | 0.61 | 0.12 |
|  |  | Female | 1.06 | (0.95 - 1.17) | 0.29 | 3 | 10,174 | 0.08 |  |
| rs3817198 | Breast cancer | Male | 0.96 | (0.85 - 1.08) | 0.46 | 3 | 8,223 | 0.41 | **0.02** |
|  |  | Female | 1.14 | (1.04 - 1.24) | **4.01E-03** | 4 | 13,886 | 0.38 |  |
| rs401681 | Lung cancer | Male | 0.89 | (0.8 - 0.99) | **0.03** | 3 | 8,228 | 0.92 | 0.65 |
|  |  | Female | 0.86 | (0.79 - 0.94) | **4.29E-04** | 4 | 13,881 | 0.46 |  |
| rs402710 | Lung cancer | Male | 0.83 | (0.73 - 0.95) | **0.01** | 2 | 5,802 | 0.56 | 0.37 |
|  |  | Female | 0.9 | (0.81 - 0.99) | **0.04** | 3 | 10,189 | 0.05 |  |
| rs4132601 | Leukemia (ALL) | Male | 1 | (0.82 - 1.21) | 0.97 | 2 | 3,454 | 0.89 | 0.95 |
|  |  | Female | 0.99 | (0.89 - 1.1) | 0.83 | 3 | 9,883 | 0.22 |  |
| rs4242382 | Prostate cancer | Male | 0.96 | (0.71 - 1.28) | 0.77 | 2 | 3,426 | 0.34 | 0.86 |
|  |  | Female | 0.99 | (0.84 - 1.16) | 0.86 | 3 | 9,872 | 0.81 |  |
| rs4254535 | Lung cancer | Male | 0.93 | (0.79 - 1.08) | 0.34 | 1 | 4,793 | . | 0.34 |
|  |  | Female | 1.02 | (0.91 - 1.14) | 0.77 | 2 | 9,283 | 0.19 |  |
| rs4295627 | Glioma | Male | 1.04 | (0.72 - 1.52) | 0.82 | 1 | 1,024 | . | 0.70 |
|  |  | Female | 0.96 | (0.82 - 1.13) | 0.66 | 2 | 6,194 | 0.92 |  |
| SNP | Previous trait association | Sex | OR | 95% CI | P-value | # studies | n | Study P-heterogeneity | Sex P-heterogeneity |
| rs4324715 | Testicular cancer | Male | 0.94 | (0.79 - 1.11) | 0.44 | 2 | 3,428 | 0.85 | 0.60 |
|  |  | Female | 0.99 | (0.9 - 1.08) | 0.77 | 3 | 9,862 | 0.02 |  |
| rs4324798 | Lung cancer | Male | 1.04 | (0.85 - 1.27) | 0.69 | 3 | 8,109 | 0.02 | 0.98 |
|  |  | Female | 1.04 | (0.9 - 1.2) | 0.61 | 4 | 13,688 | 0.34 |  |
| rs4415084 | Breast cancer | Male | 1.04 | (0.91 - 1.2) | 0.56 | 1 | 4,692 | . | 0.48 |
|  |  | Female | 0.98 | (0.88 - 1.08) | 0.68 | 2 | 9,236 | 0.89 |  |
| rs4430796 | Prostate cancer | Male | 0.97 | (0.85 - 1.1) | 0.59 | 2 | 5,795 | 0.51 | 0.14 |
|  |  | Female | 1.09 | (0.99 - 1.2) | 0.08 | 3 | 10,186 | 0.64 |  |
| rs4444235 | Colorectal cancer | Male | 1.05 | (0.92 - 1.19) | 0.47 | 2 | 5,813 | 0.58 | 0.79 |
|  |  | Female | 1.03 | (0.93 - 1.13) | 0.61 | 3 | 10,197 | 0.16 |  |
| rs445114 | Prostate cancer | Male | 1.12 | (0.84 - 1.5) | 0.45 | 1 | 1,012 | . | 0.59 |
|  |  | Female | 1.03 | (0.91 - 1.16) | 0.67 | 2 | 6,185 | 0.13 |  |
| rs4474514 | Testicular cancer | Male | 1.11 | (0.89 - 1.39) | 0.36 | 2 | 3,448 | 0.34 | 0.59 |
|  |  | Female | 1.04 | (0.92 - 1.17) | 0.56 | 3 | 9,879 | 0.81 |  |
| rs458685 | Breast cancer | Male | 1.06 | (0.9 - 1.24) | 0.48 | 2 | 7,220 | 0.78 | 0.84 |
|  |  | Female | 1.03 | (0.88 - 1.21) | 0.68 | 2 | 7,700 | 0.46 |  |
| rs4624820 | Testicular germ cell tumor | Male | 0.85 | (0.72 - 1.01) | 0.07 | 2 | 3,447 | 0.81 | 0.23 |
|  |  | Female | 0.96 | (0.87 - 1.06) | 0.41 | 3 | 9,877 | 0.04 |  |
| rs4657482 | Testicular germ cell tumor | Male | 1.11 | (0.82 - 1.48) | 0.50 | 1 | 1,026 | . | 0.45 |
|  |  | Female | 0.98 | (0.86 - 1.11) | 0.73 | 2 | 6,182 | 0.60 |  |
| rs4699052 | Testicular germ cell tumor | Male | 1.06 | (0.79 - 1.42) | 0.68 | 1 | 1,021 | . | 0.49 |
|  |  | Female | 0.95 | (0.84 - 1.07) | 0.42 | 2 | 6,179 | 0.61 |  |
| rs4779584 | Colorectal cancer | Male | 0.99 | (0.86 - 1.14) | 0.87 | 3 | 8,240 | 0.33 | 0.46 |
|  |  | Female | 0.93 | (0.83 - 1.03) | 0.16 | 4 | 13,890 | 0.39 |  |
| rs4782780 | Prostate cancer | Male | 0.93 | (0.83 - 1.05) | 0.27 | 2 | 7,222 | 0.62 | 0.17 |
|  |  | Female | 1.05 | (0.93 - 1.19) | 0.41 | 2 | 7,701 | 0.39 |  |
| SNP | Previous trait association | Sex | OR | 95% CI | P-value | # studies | n | Study P-heterogeneity | Sex P-heterogeneity |
| rs4809324 | Glioma (high-grade) | Male | . | . | . | 0 | 0 | . | . |
|  |  | Female | 1.05 | (0.86 - 1.28) | 0.62 | 1 | 5,276 | . |  |
| rs4857841 | Prostate cancer | Male | 0.88 | (0.78 - 1) | 0.05 | 3 | 8,232 | 0.24 | 0.05 |
|  |  | Female | 1.03 | (0.94 - 1.13) | 0.55 | 4 | 13,861 | 0.86 |  |
| rs4939827 | Colorectal cancer | Male | 0.92 | (0.83 - 1.03) | 0.15 | 3 | 8,221 | 0.64 | 0.33 |
|  |  | Female | 0.99 | (0.91 - 1.08) | 0.81 | 4 | 13,884 | 0.50 |  |
| rs4961199 | Prostate cancer | Male | 0.91 | (0.78 - 1.06) | 0.22 | 3 | 8,235 | 0.61 | 0.14 |
|  |  | Female | 1.05 | (0.93 - 1.17) | 0.44 | 4 | 13,889 | 0.88 |  |
| rs4962416 | Prostate cancer | Male | 0.96 | (0.85 - 1.08) | 0.47 | 3 | 8,232 | 0.81 | 0.36 |
|  |  | Female | 1.03 | (0.94 - 1.12) | 0.58 | 4 | 13,893 | 0.19 |  |
| rs4973768 | Breast cancer | Male | 0.95 | (0.85 - 1.06) | 0.37 | 3 | 8,234 | 0.12 | 0.92 |
|  |  | Female | 0.96 | (0.88 - 1.04) | 0.31 | 4 | 13,885 | 0.44 |  |
| rs4975616 | Lung cancer | Male | 0.87 | (0.78 - 0.97) | **0.02** | 3 | 8,247 | 0.93 | 0.86 |
|  |  | Female | 0.86 | (0.79 - 0.94) | **5.69E-04** | 4 | 13,888 | 0.54 |  |
| rs4977756 | Glioma | Male | 0.96 | (0.81 - 1.15) | 0.68 | 2 | 3,455 | 0.86 | 0.95 |
|  |  | Female | 0.97 | (0.88 - 1.07) | 0.53 | 3 | 9,888 | 0.55 |  |
| rs498872 | Glioma | Male | 1.2 | (1 - 1.44) | 0.05 | 2 | 3,447 | 0.94 | 0.11 |
|  |  | Female | 1 | (0.88 - 1.14) | 1.00 | 2 | 6,182 | 0.11 |  |
| rs505922 | Pancreatic cancer | Male | 0.83 | (0.69 - 1) | **0.05** | 2 | 3,455 | 0.23 | 0.38 |
|  |  | Female | 0.91 | (0.83 - 1.01) | 0.08 | 3 | 9,884 | 0.58 |  |
| rs5759167 | Prostate cancer | Male | 0.95 | (0.84 - 1.08) | 0.46 | 2 | 5,787 | 0.08 | 0.41 |
|  |  | Female | 1.04 | (0.89 - 1.21) | 0.66 | 2 | 4,894 | 0.02 |  |
| rs5945572 | Prostate cancer | Male | 1 | (0.91 - 1.09) | 0.92 | 2 | 5,820 | 0.28 | 0.93 |
|  |  | Female | 1 | (0.91 - 1.11) | 0.98 | 3 | 10,194 | 0.91 |  |
| rs5945619 | Prostate cancer | Male | 1 | (0.91 - 1.1) | 0.98 | 2 | 5,785 | 0.28 | 0.92 |
|  |  | Female | 1.01 | (0.91 - 1.12) | 0.87 | 3 | 10,156 | 0.94 |  |
| SNP | Previous trait association | Sex | OR | 95% CI | P-value | # studies | n | Study P-heterogeneity | Sex P-heterogeneity |
| rs6001749 | Prostate cancer | Male | 1.09 | (0.89 - 1.33) | 0.41 | 2 | 3,436 | 0.89 | 0.54 |
|  |  | Female | 1 | (0.83 - 1.2) | 1.00 | 2 | 4,594 | 0.08 |  |
| rs6010620 | Glioma | Male | 0.99 | (0.73 - 1.36) | 0.96 | 1 | 1,009 | . | 0.94 |
|  |  | Female | 1.01 | (0.88 - 1.16) | 0.92 | 2 | 6,178 | 0.40 |  |
| rs620861 | Prostate cancer | Male | 1.11 | (0.97 - 1.26) | 0.12 | 2 | 5,817 | 0.84 | 0.79 |
|  |  | Female | 1.08 | (0.92 - 1.26) | 0.35 | 2 | 4,922 | 0.25 |  |
| rs630014 | Pancreatic cancer | Male | 0.91 | (0.77 - 1.08) | 0.30 | 2 | 3,440 | 0.59 | 0.31 |
|  |  | Female | 1.01 | (0.92 - 1.11) | 0.83 | 3 | 9,884 | 0.72 |  |
| rs6435862 | Neuroblastoma (high-risk) | Male | 1.09 | (0.9 - 1.32) | 0.36 | 2 | 3,380 | 0.85 | 0.22 |
|  |  | Female | 0.95 | (0.85 - 1.06) | 0.37 | 3 | 9,874 | 0.27 |  |
| rs6457327 | NHL (Follicular lymphoma) | Male | 0.85 | (0.71 - 1.02) | 0.08 | 2 | 3,452 | 0.83 | 0.12 |
|  |  | Female | 1 | (0.91 - 1.11) | 0.95 | 3 | 9,885 | 0.68 |  |
| rs6465657 | Prostate cancer | Male | 1.1 | (0.98 - 1.22) | 0.10 | 3 | 8,241 | 0.67 | **0.04** |
|  |  | Female | 0.95 | (0.88 - 1.03) | 0.24 | 4 | 13,886 | 0.63 |  |
| rs6504950 | Breast cancer | Male | 0.98 | (0.87 - 1.11) | 0.76 | 3 | 8,220 | 0.17 | 0.70 |
|  |  | Female | 0.95 | (0.87 - 1.04) | 0.29 | 4 | 13,884 | 0.29 |  |
| rs6556756 | Breast cancer | Male | 0.88 | (0.73 - 1.06) | 0.19 | 2 | 7,219 | 0.23 | 0.66 |
|  |  | Female | 0.93 | (0.81 - 1.06) | 0.27 | 3 | 12,978 | 0.70 |  |
| rs671 | Esophageal cancer | Male | 0 | . | 0.99 | 1 | 1,029 | . | 1.00 |
|  |  | Female | 0 | . | 0.99 | 1 | 6,192 | . |  |
| rs6939340 | Neuroblastoma (high-risk) | Male | 0.76 | (0.57 - 1.02) | 0.07 | 1 | 1,000 | . | **0.03** |
|  |  | Female | 1.08 | (0.96 - 1.21) | 0.22 | 2 | 6,177 | 0.48 |  |
| rs6983267 | Colorectal cancer | Male | 1.07 | (0.96 - 1.2) | 0.21 | 3 | 8,220 | 0.73 | 0.80 |
|  |  | Female | 1.05 | (0.97 - 1.14) | 0.22 | 4 | 13,877 | 0.61 |  |
| rs6983561 | Prostate cancer | Male | 0.82 | (0.37 - 1.81) | 0.62 | 1 | 1,026 | . | 0.91 |
|  |  | Female | 0.86 | (0.62 - 1.2) | 0.37 | 2 | 6,190 | 0.35 |  |
| SNP | Previous trait association | Sex | OR | 95% CI | P-value | # studies | n | Study P-heterogeneity | Sex P-heterogeneity |
| rs7000448 | Prostate cancer | Male | 1.04 | (0.93 - 1.16) | 0.51 | 3 | 8,231 | 0.25 | 0.74 |
|  |  | Female | 1.07 | (0.95 - 1.2) | 0.26 | 3 | 8,602 | 0.08 |  |
| rs7014346 | Colorectal cancer | Male | 1.03 | (0.92 - 1.15) | 0.59 | 3 | 8,219 | 0.93 | 0.92 |
|  |  | Female | 1.02 | (0.94 - 1.12) | 0.58 | 4 | 13,878 | 0.30 |  |
| rs7089424 | Leukemia (ALL) | Male | 1.1 | (0.83 - 1.47) | 0.51 | 1 | 1,024 | . | 0.73 |
|  |  | Female | 1.04 | (0.92 - 1.18) | 0.51 | 2 | 6,188 | 0.88 |  |
| rs710521 | Urinary bladder cancer | Male | 1.16 | (0.95 - 1.41) | 0.16 | 2 | 3,444 | 0.64 | 0.79 |
|  |  | Female | 1.12 | (0.98 - 1.28) | 0.10 | 2 | 6,164 | 0.17 |  |
| rs7117034 | Prostate cancer | Male | 1.26 | (1.09 - 1.45) | **2.07E-03** | 2 | 5,775 | 0.20 | 0.62 |
|  |  | Female | 1.18 | (0.99 - 1.42) | 0.07 | 2 | 4,900 | 0.44 |  |
| rs7127900 | Prostate cancer | Male | 0.96 | (0.67 - 1.39) | 0.83 | 1 | 1,011 | . | 0.96 |
|  |  | Female | 0.97 | (0.84 - 1.13) | 0.69 | 2 | 6,182 | 0.64 |  |
| rs7176508 | Non-Hodgkin lymphoma (CLL) | Male | 1.08 | (0.87 - 1.35) | 0.48 | 1 | 2,428 | . | 0.87 |
|  |  | Female | 1.1 | (1 - 1.22) | 0.05 | 2 | 8,967 | 0.49 |  |
| rs719725 | Colorectal cancer | Male | 0.94 | (0.84 - 1.05) | 0.29 | 3 | 8,181 | 0.18 | **0.03** |
|  |  | Female | 1.11 | (1.01 - 1.21) | **0.02** | 4 | 13,860 | 0.92 |  |
| rs721048 | Prostate cancer | Male | 1.31 | (0.92 - 1.86) | 0.13 | 1 | 1,026 | . | 0.23 |
|  |  | Female | 1.04 | (0.89 - 1.21) | 0.62 | 2 | 6,175 | 0.22 |  |
| rs735665 | Non-Hodgkin lymphoma (CLL) | Male | 1.14 | (0.92 - 1.41) | 0.22 | 2 | 3,444 | 0.75 | 0.16 |
|  |  | Female | 0.96 | (0.84 - 1.08) | 0.49 | 3 | 9,879 | 0.15 |  |
| rs748404 | Lung cancer | Male | 1.08 | (0.93 - 1.25) | 0.32 | 2 | 7,176 | 0.25 | 0.42 |
|  |  | Female | 1 | (0.9 - 1.11) | 1.00 | 3 | 12,923 | 0.66 |  |
| rs7501939 | Prostate cancer | Male | 1 | (0.88 - 1.14) | 0.98 | 2 | 5,802 | 0.99 | 0.97 |
|  |  | Female | 1 | (0.91 - 1.11) | 0.93 | 3 | 10,183 | 0.92 |  |
| rs7538876 | Basal cell carcinoma | Male | 1.02 | (0.85 - 1.22) | 0.82 | 2 | 3,450 | 0.52 | 0.82 |
|  |  | Female | 1.05 | (0.95 - 1.15) | 0.36 | 3 | 9,877 | 0.95 |  |
| SNP | Previous trait association | Sex | OR | 95% CI | P-value | # studies | n | Study P-heterogeneity | Sex P-heterogeneity |
| rs757978 | Non-Hodgkin lymphoma (CLL) | Male | 1.04 | (0.74 - 1.47) | 0.82 | 1 | 2,428 | . | 0.51 |
|  |  | Female | 0.91 | (0.77 - 1.08) | 0.30 | 2 | 8,971 | 0.01 |  |
| rs7626795 | Lung cancer | Male | 0.81 | (0.66 - 0.99) | **0.04** | 2 | 5,793 | 0.24 | 0.11 |
|  |  | Female | 0.98 | (0.87 - 1.11) | 0.77 | 4 | 13,850 | 0.27 |  |
| rs7679673 | Prostate cancer | Male | 0.99 | (0.87 - 1.13) | 0.89 | 2 | 5,765 | 0.21 | 0.56 |
|  |  | Female | 0.94 | (0.86 - 1.04) | 0.25 | 3 | 10,143 | 0.93 |  |
| rs7809758 | Leukemia (ALL) | Male | 0.98 | (0.82 - 1.17) | 0.81 | 2 | 3,453 | 0.93 | 0.99 |
|  |  | Female | 0.98 | (0.89 - 1.08) | 0.69 | 3 | 9,884 | 0.09 |  |
| rs7837688 | Prostate cancer | Male | 0.84 | (0.67 - 1.06) | 0.15 | 2 | 5,819 | 0.35 | 0.27 |
|  |  | Female | 0.99 | (0.84 - 1.16) | 0.87 | 3 | 10,199 | 0.84 |  |
| rs7841060 | Prostate cancer | Male | 0.92 | (0.78 - 1.08) | 0.31 | 2 | 5,787 | 0.00 | 0.14 |
|  |  | Female | 1.07 | (0.95 - 1.2) | 0.27 | 3 | 10,183 | 0.87 |  |
| rs7931342 | Prostate cancer | Male | 1.05 | (0.89 - 1.25) | 0.56 | 2 | 3,451 | 0.76 | 0.78 |
|  |  | Female | 1.02 | (0.93 - 1.13) | 0.63 | 3 | 9,885 | 0.37 |  |
| rs801114 | Basal cell carcinoma | Male | 0.94 | (0.78 - 1.12) | 0.47 | 2 | 3,455 | 0.96 | 0.45 |
|  |  | Female | 1.01 | (0.92 - 1.12) | 0.79 | 3 | 9,885 | 0.96 |  |
| rs8034191 | Lung cancer | Male | 0.79 | (0.65 - 0.95) | **0.01** | 2 | 3,451 | 0.40 | **0.01** |
|  |  | Female | 1.03 | (0.93 - 1.14) | 0.54 | 3 | 9,884 | 0.51 |  |
| rs8042374 | Lung cancer | Male | 1.01 | (0.89 - 1.15) | 0.87 | 3 | 8,183 | 0.00 | 0.36 |
|  |  | Female | 0.94 | (0.85 - 1.03) | 0.20 | 4 | 13,849 | 0.71 |  |
| rs8102476 | Prostate cancer | Male | 1.05 | (0.92 - 1.19) | 0.46 | 2 | 5,817 | 0.54 | 0.34 |
|  |  | Female | 0.95 | (0.82 - 1.11) | 0.54 | 2 | 4,926 | 0.23 |  |
| rs872071 | Non-Hodgkin lymphoma (CLL) | Male | 1.08 | (0.81 - 1.43) | 0.60 | 1 | 1,026 | . | 0.99 |
|  |  | Female | 1.08 | (0.96 - 1.21) | 0.22 | 2 | 6,184 | 0.98 |  |
| rs889312 | Breast cancer | Male | 1.02 | (0.91 - 1.15) | 0.71 | 3 | 8,214 | 0.07 | 0.68 |
|  |  | Female | 1.06 | (0.96 - 1.16) | 0.24 | 4 | 13,884 | 0.78 |  |
| SNP | Previous trait association | Sex | OR | 95% CI | P-value | # studies | n | Study P-heterogeneity | Sex P-heterogeneity |
| rs9295740 | Lung cancer | Male | 0.93 | (0.8 - 1.07) | 0.30 | 3 | 8,248 | 0.24 | 0.91 |
|  |  | Female | 0.92 | (0.82 - 1.02) | 0.12 | 4 | 13,886 | 0.66 |  |
| rs931794 | Lung cancer | Male | 0.88 | (0.78 - 0.99) | **0.03** | 3 | 8,211 | 0.08 | 0.06 |
|  |  | Female | 1.01 | (0.93 - 1.1) | 0.83 | 4 | 13,830 | 0.81 |  |
| rs9364554 | Prostate cancer | Male | 1.08 | (0.96 - 1.21) | 0.21 | 3 | 8,241 | 0.01 | 0.24 |
|  |  | Female | 0.99 | (0.9 - 1.08) | 0.76 | 4 | 13,876 | 0.26 |  |
| rs944289 | Thyroid cancer | Male | 0.96 | (0.72 - 1.27) | 0.75 | 1 | 1,029 | . | 0.68 |
|  |  | Female | 1.02 | (0.92 - 1.12) | 0.74 | 3 | 9,802 | 0.25 |  |
| rs9543325 | Pancreatic cancer | Male | 0.89 | (0.71 - 1.12) | 0.32 | 1 | 2,428 | . | 0.79 |
|  |  | Female | 0.92 | (0.83 - 1.02) | 0.12 | 2 | 8,969 | 0.93 |  |
| rs961253 | Colorectal cancer | Male | 1 | (0.9 - 1.12) | 0.95 | 3 | 8,234 | 0.60 | 0.64 |
|  |  | Female | 1.04 | (0.95 - 1.13) | 0.39 | 4 | 13,885 | 0.31 |  |
| rs9623117 | Prostate cancer | Male | 1.04 | (0.91 - 1.2) | 0.56 | 2 | 7,191 | 0.74 | 0.60 |
|  |  | Female | 0.99 | (0.9 - 1.1) | 0.91 | 3 | 12,975 | 0.70 |  |
| rs9642880 | Urinary bladder cancer | Male | 1.01 | (0.81 - 1.26) | 0.92 | 1 | 2,428 | . | 0.67 |
|  |  | Female | 0.96 | (0.87 - 1.06) | 0.41 | 2 | 8,968 | 0.76 |  |
| rs965513 | Thyroid cancer | Male | 1.06 | (0.88 - 1.27) | 0.56 | 2 | 3,450 | 0.22 | 0.41 |
|  |  | Female | 0.97 | (0.87 - 1.07) | 0.51 | 3 | 9,880 | 0.56 |  |
| rs981782 | Breast cancer | Male | 0.95 | (0.84 - 1.08) | 0.41 | 2 | 5,804 | 0.45 | 0.74 |
|  |  | Female | 0.97 | (0.89 - 1.07) | 0.60 | 3 | 10,200 | 0.28 |  |
| rs9929218 | Colorectal cancer | Male | 0.97 | (0.86 - 1.09) | 0.57 | 3 | 8,250 | 0.34 | 0.78 |
|  |  | Female | 0.95 | (0.86 - 1.04) | 0.23 | 4 | 13,890 | 0.66 |  |
| rs995030 | Testicular germ cell tumor | Male | 1.15 | (0.91 - 1.45) | 0.24 | 2 | 3,446 | 0.28 | 0.47 |
|  |  | Female | 1.04 | (0.92 - 1.18) | 0.50 | 3 | 9,881 | 0.82 |  |
| rs999737 | Breast cancer | Male | 0.88 | (0.75 - 1.04) | 0.13 | 1 | 4,758 | . | 0.13 |
|  |  | Female | 1.03 | (0.92 - 1.16) | 0.62 | 2 | 9,218 | 0.98 |  |
